# Amine-Appended Hyper-Crosslinked Polymers for Direct Air Capture of CO_2_

**DOI:** 10.1021/acssuschemeng.5c08715

**Published:** 2026-01-17

**Authors:** Tristan L. Spreng, David Danaci, Preshti D. Ram, Daryl R. Williams, Ronny Pini, Camille Petit

**Affiliations:** † Department of Chemical Engineering, 4615 Imperial College London, London SW7 2AZ, U.K.; ‡ The Sargent Centre for Process Systems Engineering, Imperial College London, London SW7 2AZ, U.K.; § I-X Centre for AI in Science, Imperial College London, London W12 0BZ, U.K.

**Keywords:** direct air capture, hyper-crosslinked polymers, polymeric adsorbents, carbon dioxide, adsorption, sorption equilibrium, diffusion, amine efficiency

## Abstract

Capturing CO_2_ from the ambient atmosphere is a promising method to reduce the impact of climate change. Fast deployment and scale-up of adsorption-based direct air capture (DAC) technologies are needed to meet the IPCC target and rely, in part, on the development of efficient and scalable low-cost adsorbents. While a benchmark DAC adsorbent, the polymeric resin Lewatit VP OC 1065, has been established, the reasons behind its performance and the potential for further optimization remain largely unknown. Indeed, a fundamental understanding of the relationship between adsorbent pore structure, chemistry, and DAC performance, both equilibrium and kinetics, has yet to be formulated. Here, we have built on the chemistry of Lewatit and synthesized a hyper-crosslinked polymer (HCP) by grafting a microporous chlorine-functionalized support with diethylenetriamine. We produced four different adsorbents by varying the polymerization duration between 10 min and 19 h to assess the impact of pore structure on CO_2_ uptake at 400 ppm. Reduced degrees of polymerization (i.e., shorter polymerization durations) resulted in higher accessible micropore volume and consequentially increased CO_2_ uptake and amine efficiency. The best sample achieved an equilibrium uptake of 0.43 mmol/g (400 ppm of CO_2_, 298 K), which is about half that of the benchmark adsorbent Lewatit VP OC 1065. We have then assessed the CO_2_ sorption kinetics of this sample (grain size of 24–74 μm) at 400 ppm and 303 K using a gravimetric technique and have compared the results to those of other amine-grafted polymeric adsorbents. We measured a lower bound linear driving force constant (*k*_LDF_) of 0.0120 ± 0.0004 s^–1^. This value is 5.5 times faster than that of the benchmark adsorbent Lewatit VP OC 1065 with the same grain size of 24–74 μm, highlighting the importance of macropore diffusion in addition to the CO_2_ reaction kinetics. This study shows how synthesis operating conditions alter the pore structures and adsorption behavior of porous polymers and provides the foundation to design better and faster DAC adsorbents.

## Graphical abstract

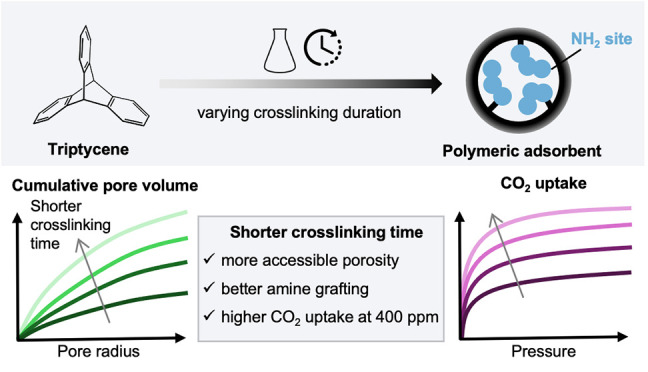

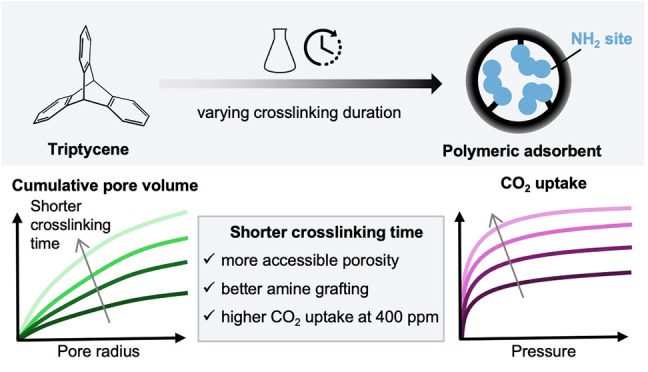

## 1 Introduction

One hundred ninety-six countries signed the Paris Agreement in 2015 to limit global warming to well below 2 °C and pursue efforts to limit the temperature increase to below 1.5 °C above preindustrial levels. The International Panel on Climate Change (IPCC) found that this goal is only attainable with carbon dioxide removal (CDR) approaches.^1^ Such approaches have a net-negative carbon footprint and are therefore an integral part of achieving net-zero by compensating for residual emissions and removing historical CO_2_ emissions.^2^

Direct air capture of CO_2_ (DAC) represents a promising CDR technology that allows a priori for gigatonne scaling and might interfere less with land and water use than nature-based solutions.^3^ If coupled with the underground storage of CO_2_, the removal is permanent. DAC allows for the straightforward quantification of CO_2_ captured, and high net CO_2_ removal if low-carbon energy is used. Despite these positive attributes, the current capacity of DAC facilities is only 17 kt CO_2_ yr^–1^, far below the required gigatonne capacity target by midcentury.^4^ A main reason for this lag is the discrepancy in carbon pricing (approximately $80 and $70 per tonne of CO_2_ within the EU and UK emissions trading systems, respectively) and the social cost of CO_2_, estimated at $185 per tonne CO_2_,^5^ compared to the cost of DAC currently estimated at approximately $1000 per tonne CO_2_ for the largest operating DAC plant.^6^ Reducing capital and operational costs through the design of DAC technologies with better performance is the key to making DAC economically viable in the future.^7^

In the context of adsorption-based DAC, the performance of the process is influenced by numerous adsorbent properties and metrics. These include but are not limited to the CO_2_ adsorption isotherm, CO_2_ adsorption kinetics, enthalpy of adsorption, H_2_O coadsorption, selectivity of CO_2_ over N_2_, adsorbent bed density, heat transfer coefficient, adsorbent heat capacity, and stability.^8,9^ The relative importance of these properties depends on the given process configuration, whereby heat, vacuum, steam, or combinations thereof are applied to regenerate the sorbent. For instance, a recent study by Young et al. found that when using steam in a Temperature-Vacuum Swing Adsorption process (TVSA), the mass transfer coefficient of CO_2_ uptake becomes a more important determinant of productivity, energy requirements, and purity of the process than equilibrium CO_2_ uptake.^9^

More than 200 DAC adsorbents were reported in the literature between 2016 and 2021, including metal-organic frameworks (MOFs), silicas, carbons, zeolites, metal oxides, and polymers.^4^ A common technique for increasing the CO_2_ uptake at low partial pressures is to attach amine groups to the internal surfaces of these porous materials. The high affinity for CO_2_ is driven by acid–base interactions between CO_2_ molecules and amine groups, leading to the formation of a covalent C–N bond, which can be reversibly broken by applying heat. The two species resulting from the adsorption of CO_2_ under dry conditions are carbamic acid or alkylammonium carbamate, and their ratio depends on the chemical environment at the adsorbent surface.^10^ Low surface grafting densities of amines favor the formation of neutral carbamic acid species, while high surface densities favor charged alkylammonium carbamate sites.^11^ This observation has implications on the so-called “amine efficiency”, as the latter species require two amine groups per adsorbed CO_2_, while carbamic acid species are formed from one amine per adsorbed CO_2_. Hydrogen bonding with adjacent amine or hydroxy groups also plays a key role in stabilizing the intermediates and products of the CO_2_ adsorption reaction.^12^ The two species formed under humid conditions are alkylammonium carbamate and alkylammonium bicarbonate, and their ratios depend on the partial pressures of H_2_O and CO_2_. While some studies highlight the preferred formation of bicarbonate under humid conditions, others find that ammonium carbamate also dominates in the presence of water.^13,14^ In addition to increasing the CO_2_ uptake capacity in polymeric resins,^15^ H_2_O coadsorption can lower the activation barrier for CO_2_ adsorption/reaction.^16^ The resulting increase in CO_2_ uptake under humid conditions is explained by preferential alkylammonium bicarbonate formation with a 1:1 stoichiometric ratio between the amine group and CO_2_ (Figure S1). A mechanistic study revealed that bicarbonate formation is initiated by nucleophilic attack of the water molecule (and not the amine) onto CO_2_ with subsequent proton rearrangement facilitated by hydrogen-bonded amine groups.^14^ Another possible mechanism for enhanced CO_2_ adsorption under humid conditions is polymer swelling, which causes pore opening.^17^

Alongside their tunable porosity, low cost, and scalability, the enhancement of adsorption under humid conditions makes polymers with amine functionalization promising DAC adsorbents.^8,18,19^ The benchmark DAC adsorbent, Lewatit VP OC 1065 (“Lewatit” herein), belongs to this class of materials and is a cross-linked polystyrene resin with primary amine functionalization with a CO_2_ uptake of 0.95 mmol/g at 0.04 kPa and 298 K.^20^ As a meso-/macroporous adsorbent, it has a relatively low Brunauer–Emmett–Teller (BET) area of ∼30 m^2^/g, and an amine content of approximately 5.9 mmol/g.^20,21^ Lewatit is originally a resin for separations from liquid process streams and is therefore unlikely to be optimized for DAC. For instance, the linear driving force (LDF) constant of CO_2_ adsorption at 400 ppm (303 K) is 3.1 × 10^–4^ s^–1^, which needs to be improved to achieve a commercially viable DAC process.^22^

Hyper-crosslinked polymers (HCPs) share a similar aromatic crosslinked backbone with Lewatit, but they achieve much higher porosity, including in the micro- to ultramicropore region.^23,24^ These materials are commonly synthesized by Friedel–Crafts crosslinking of aromatic monomers (Figure S2) composed of hydrophobic sp^2^ and sp^3^ carbon atoms with CO_2_ equilibrium uptakes around 2–3 mmol/g at 1 bar (298 K) and negligible CO_2_ uptake at 400 ppm.^25–31^ High CO_2_ uptakes at low pressures can be achieved by functionalizing amine groups onto the material surface, resulting in a chemisorbent material. However, functionalization methods such as the use of amine-containing monomers,^26,31–34^ reductive amination,^35,36^ and nitration, followed by reduction^25,37^ have so far resulted in materials with near-linear CO_2_ isotherms characteristic of physisorbents. Only the grafting of polyamine molecules^33–35,38–42^ has led to materials with significant CO_2_ uptake at low pressures.^34,36,41,42^ Another functionalization approach is impregnation, in which the polymeric support is soaked in the polyamine to establish noncovalent interactions (e.g., van der Waals forces). While these materials achieve high equilibrium uptakes of CO_2_ at low pressures, cyclic stability is generally compromised due to desorption of the physisorbed amine molecules during the regeneration step.^29,43,44^ Although several studies have looked at amine-functionalized HCPs with apparent CO_2_ chemisorbing qualities, none have reported the CO_2_ uptake at 400 ppm or a mass transfer coefficient.^34,38,41,42^

Our hypothesis is that HCPs could serve as an effective platform to systematically study the relationship between material structure/porosity, material chemistry, and DAC performance. In fact, modifications to synthesis parameters, such as the choice of starting monomers or reaction conditions, can directly impact the structural properties of the material,^27,45–47^ while the degree of amine functionalization can be tailored following the methods described above. Hence, the objective of this work is to synthesize four amine-grafted HCPs with varying degrees of polymerization and report their CO_2_ uptake values at atmospheric concentrations to investigate the relationship between pore structure and CO_2_ uptake capacity. This is complemented with the measurement of full CO_2_ isotherms up to 1 bar and skeletal densities, which is important to assess the suitability of these adsorbents for DAC. The CO_2_ uptake kinetics at 400 ppm of the highest-adsorbing sample are determined using the LDF model to allow for comparison with other benchmark adsorbents. To carry out this study, we opted for amine-grafted HCPs made from Friedel–Crafts cross-linking of triptycene. We functionalized the samples via chloromethylation with subsequent nucleophilic substitution of diethylenetriamine (DETA). We chose this polyamine, as it resulted in high CO_2_ uptakes in previous studies^41,42^ and it is small enough to functionalize micropores, in contrast to larger molecules like polyethylenimine (PEI). We synthesized four HCPs at increasing polymerization times and monitored the effect on the pore structure and sorption performance. Ultimately, elucidating the relationship between the pore structure of amine-functionalized HCPs and their equilibrium and kinetic sorption performance would allow for a framework for optimizing adsorbent performance through the systematic tuning of the synthesis parameters.

## 2 Experimental Section

### 2.1 Materials

Triptycene (>98%) was purchased from Tokyo Chemical Industry (TCI). Dimethoxymethane (99%), 1,2-dichloroethane (anhydrous, 99.8%), paraformaldehyde (powder, 95%), acetic acid (>99.8%), and diethylenetriamine (99%) were purchased from Sigma-Aldrich. Iron­(III) chloride (anhydrous, 98%) and phosphoric acid (>98%) were purchased from Thermo Scientific. Methanol (reagent grade) and concentrated hydrochloric acid (37%) were purchased from VWR. All chemicals were used as received without further purification. Triptycene (98%) for the repeat synthesis was purchased from Sigma-Aldrich and used as received.

For gas sorption measurements, CO_2_ (BOC, N5.0 grade) and N_2_ (BOC, N6.0 grade) were used. Helium (BOC, N5.0 grade) was used for the pycnometry measurements. For thermogravimetric analysis, N_2_ (BOC, N6.0 grade) and a custom-made 800 ppm CO_2_ in He mixture (BOC, CO_2_ and He, both of research grade) were used.

### 2.2 Hyper-Crosslinked Polymer Synthesis

The synthetic procedure is based on work by Li et al.^41^ and is summarized in Figure 1. Triptycene (0.64 g, 2.5 mmol) and dimethoxymethane (1.14 g, 15.0 mmol) were added to a three-neck round-bottom flask fitted with a reflux condenser. Anhydrous 1,2-dichloroethane (5 mL) was added together with iron­(III) chloride (2.44 g, 15.0 mmol), and the reaction mixture was stirred at 45 °C for [5 min, 30 min, 30 min, 5 h] and then at 80 °C for [10 min, 30 min, 2 h, 19 h] to obtain [HCP-1–10 min, HCP-1–30 min, HCP-1–2 h, HCP-1–19 h], respectively. The resulting solid was allowed to cool to room temperature, washed with 100 mL of methanol by gravity filtration, broken up into smaller pieces using a spatula, and Soxhlet extracted with methanol for 24 h. A brown solid (HCP-1) was obtained after being dried in vacuo at 60 °C for at least 12 h with a yield of 33 to 49%, assuming six dimethoxymethane linkers per triptycene molecule. A mixture of 0.30 g HCP-1, 1.50 g paraformaldehyde, 9.0 mL acetic acid, 4.5 mL phosphoric acid, and 30.0 mL concentrated hydrochloric acid was charged in a round-bottom flask and heated to 90 °C under reflux for 3 days. The solid was allowed to cool to room temperature and washed with 100 mL of DI water and 200 mL of methanol by gravity filtration. HCP-Cl was obtained after drying in vacuo at 60 °C for at least 12 h. Amine-functionalization was achieved by charging 0.10 g of HCP-Cl and 10.0 mL of diethylenetriamine (DETA) in a round-bottomed flask and heating at 90 °C under reflux for 3 days. The solid was cooled to room temperature and washed with 150 mL of methanol by gravity filtration to afford HCP-DETA after drying in vacuo at 60 °C for at least 12 h. We note that while the benchmark solvent dichloroethane was used for the HCP synthesis, biobased solvents and solvent-free mechanochemical routes offer sustainable alternative synthesis pathways.^48,49^

**Figure 1:**
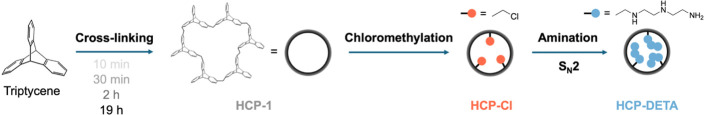
Reaction scheme of the hyper-crosslinked polymer synthesis. Black hollow circles indicate the stages of functionalization of the intermediate products. The polymers synthesized in this study differ in their crosslinking duration, which is displayed in the first step. This figure is adapted from^41^ which is available open-source under a Creative Commons (CC) Attribution 4.0 International License.

### 2.3 Methods

#### 2.3.1 Textural and Morphological Features

N_2_ sorption measurements were carried out on a volumetric analyzer (3Flex, Micromeritics) at 77 K. Samples were degassed under vacuum ex situ at 393 K and 0.002 kPa for at least 12 h (VacPrep, Micromeritics) and then degassed in situ at 393 K and 0.00002 kPa for 4 h. We have used this outgassing temperature as it was described to be suitable for the regeneration of amine-functionalized polymers in previous literature.^20,50^ We only increased the temperature to 120 °C once a vacuum below 10^‑2^ kPa was reached, and the sample was kept under nitrogen when transferred onto the instrument after outgassing. These precautions were taken to keep oxidative degradation to a minimum. There was no visible change in the appearance of the materials before and after the overnight outgassing step. Approximately 100 mg of the dried sample was used for each measurement. An equilibration condition of pressure changes less than 0.01% within a 300 s interval for pressures below 0.01 p/p° and 200 s above 0.01 p/p° was used. The total duration of measurements ranged between 12 h and a few days. The isotherms were analyzed using the Brunauer–Emmett–Teller (BET) method to determine the specific BET equivalent area using the BETSI open-source program.^51^ Pore size distribution analysis was carried out using a 2D-NLDFT algorithm that assumes slit pores below 2 nm and cylindrical pores for larger pore diameters.^52^ The cumulative pore distribution obtained from this method was used to determine pore volumes of the micropores (<2 nm) and mesopores (2–50 nm). A comparison of the experimentally obtained and 2D-NLDFT fitted isotherm is given in Figure S3.

The morphology of hyper-crosslinked polymers was investigated via scanning electron microscopy (SEM) using a JEOL JSM-6010LA with 20 kV accelerating voltage and 15 mm working distance. The samples were fixed to carbon tape and sputter coated with a 10 nm thick chromium layer. The skeletal density was determined using He pycnometry (AccuPyc II 1340, Micromeritics). Measurements were performed at 298 K with 20 preliminary purges and 10 measurement cycles. The equilibration rate was set to 34 Pa/min. The average values of the 10 measurement cycles are reported along with the standard error. The skeletal density was measured only for samples for which at least 50 mg of material was available.

#### 2.3.2 Chemical Features

Functional groups were characterized using Fourier-transform infrared spectroscopy (FTIR) using an Agilent Cary 630 spectrometer. Samples were degassed under a vacuum (<5 × 10^–3^ kPa) at 393 K for at least 12 h prior to the measurement. A total of 32 background scans and 32 sample scans over a range of 400–4000 cm^–1^ with a resolution of 1 cm^–1^ were obtained per spectrum. The data was analyzed in OriginLab Pro, and curves were smoothed via adjacent-averaging over 20 points.

The surface chemistry of the synthesized materials was analyzed using both CHNS elemental analysis and X-ray photoelectron spectroscopy (XPS) on a ThermoFisher K-Alpha instrument with a monochromatic Al–Kα X-ray source. Prior to XPS analyses, the samples were affixed to the sample holders using conductive double-sided carbon tape. The samples were degassed under a high vacuum (<5 × 10^–5^ kPa) for 30 min before conducting the XPS analysis. An X-ray spot size of 400 μm was used, and the Ar flood gun was turned on during operation. Survey scans were performed with a pass energy of 200 eV, a step size of 0.5 eV, and a dwell time of 75 ms (25 × 3 scans). Scans of the N 1s peak were performed with a pass energy of 35 eV, a step size of 0.1 eV, and a dwell time of 2.5 s (50 ms × 50 scans). The data was analyzed with the Avantage (ThermoScientific) software, and a ″smart″ background was used in combination with Voigt curves (Lorentzian/Gaussian mix of 0.2) to deconvolute the N 1s peaks. The binding energy scale was corrected for static charging using BE = 284.8 eV for the C 1s peak.^53^ The full width at half-maximum (FWHM) of component peaks was constrained to be equal. The CHNS elemental analyses were carried out externally by MEDAC Ltd. via dual determination, and the results are quoted as the mean and standard error of the atomic percentage. The analytical method employs dynamic flash combustion to achieve complete and instantaneous oxidation of the sample, converting all of the organic and inorganic constituents into their corresponding combustion products. The resulting gases pass through a reduction furnace and are transported by helium carrier gas into a chromatographic column, where they are separated into nitrogen, carbon dioxide, water, and sulfur dioxide. These species are quantified using a thermal conductivity detector that provides signals proportional to their concentrations. Instrument calibration is performed using certified reference compounds.

#### 2.3.3 Gas Equilibrium Uptake Measurements

CO_2_ and N_2_ sorption isotherms at 298 K were obtained using the same instrument and sample activation procedure as described above for the N_2_ sorption isotherms at 77 K. Equilibria conditions of a pressure change smaller than 0.01% in intervals of 600 s below 0.1 kPa and 300 s above 0.1 kPa were used. Total measurement times for the CO_2_ isotherms were between 12 h and 3 d. N_2_ isotherm measurement times were below 12 h. The temperature was maintained with a circulating thermal bath and confirmed with a total immersion thermometer before measurement.

Water uptake measurements were performed using the DVS (Dynamic Vapor Sorption) Resolution, a high-resolution gravimetric sorption analyzer developed by Surface Measurement Systems (SMS). Sample masses between 14 and 16.5 mg were used to ensure the experiments could be completed within a reasonable time frame while still providing sufficient sensitivity for accurately measuring vapor uptake. Water sorption experiments from 0 to 95% relative humidity (RH) at 298 K were completed, where the relative humidity was increased stepwise to the following concentrations: 0, 10, 20, 30, 40, 50, 60, 70, 80, 90, and 95% RH. A constant flow rate of 200 mL/min of nitrogen (N_2_) was used as the carrier gas throughout the experiment. All sorption steps operated in “dm/dt mode”, with the equilibration value set as dm/dt <0.002%/min for a period of at least 10 min. The maximum stage time of these steps was set to 360 min, after which the adsorbed amount was recorded, and the measurement moved on to the next point.

#### 2.3.4 Gas Sorption Kinetic Measurements

Kinetic sorption measurements were carried out using a NETZSCH TG 209 *F1* Libra thermogravimetric analyzer (TGA) following a method adapted from our previous work.^22^ A constant gas flow rate of 400 mL/min was used for the measurement. Between 10 and 20 mg of the sample was sieved with a 200 mesh (74 μm aperture), and particles passing through the sieve were placed into a custom-made 7 mm-diameter cylindrical basket made from a stainless steel 500 mesh (25 μm aperture). Very fine HCP dust was removed from the sample by flowing nitrogen over the closed basket at a high flow rate (>1 L/min) for 5 min. The basket was then placed into the TGA furnace and exposed to a heating ramp (5 K/min) from ambient temperature to 393 K and then held for 3 h under pure He flow while recording the weight. The temperature was then ramped down to 303 at 5 K/min and held for a further 30 min under pure He flow to obtain a background signal. The flow was then switched so that 50% of the initial He flow was replaced by 800 ppm of CO_2_ in the He mixture (BOC) to achieve a 400 ppm CO_2_ atmosphere within the sample chamber. This flow rate was held constant for at least 2 h at 303 K to achieve equilibrium. A Li-Cor LI-850 gas analyzer was connected to the TGA outlet to monitor the CO_2_ concentration. The measurement files were corrected with blank runs, which included the same protocol but only switched to pure He flow instead of a 400 ppm CO_2_ atmosphere, to correct for buoyancy effects within the TGA (Figure S4). The TGA uptake curve was converted into fractional uptake upon dividing by the equilibrium CO_2_ uptake

1 $$y\left(t\right)=\frac{m\left(t\right)-m_{initial}}{m_{final}-m_{initial}}$$

where *m*_initial_ is the weight recorded when the atmosphere of 400 ppm CO_2_ is introduced, and *m*_final_ is the weight at equilibrium, which was obtained by averaging the weight over the final 30 min of the experiment. This curve was subsequently fit to the linear driving force (LDF) model given by

2 $$f\left(t\right)=1-e^{-k_{LDF}t}$$

where *k*_LDF_ is the kinetic parameter. A maximum-likelihood estimator (MLE) was used to obtain the most probable fit, given the observed data. This is because the data indicated variability in the experimental standard deviation that was not well captured by traditional least-squares fitting. Unlike least-squares methods, MLE allows the standard deviation to be estimated as a parameter, accounting for this uncertainty. MLE is a statistical method used to estimate model parameters by maximizing the likelihood function, which maximizes the probability of the fitted parameters reproducing the observed data. The likelihood function, *L*(θ), for *N* observations is expressed as

3 $$L\left(\theta \right)=\prod_{i=1}^{N}\frac{1}{\sqrt{2\pi \sigma^{2}}}\exp\left(-\frac{{\left(y_{i}-f\left(t_{i},k_{LDF}\right)\right)}^{2}}{2\sigma^{2}}\right)$$

where *y*_*i*_ represents the experimental data, *f*(*t*_*i*_, *k*_LDF_) is the LDF model prediction at time *t*_*i*_, and σ is the standard deviation of measurement noise. The negative log-likelihood function (Φ) to be minimized is given by

4 $$\Phi =\frac{N}{2}\ln\left(2\pi \right)+\frac{1}{2}\sum_{i=1}^{N}\left[\ln\left(\sigma^{2}\right)+\frac{{\left(y_{i}-f\left(t_{i},k_{LDF}\right)\right)}^{2}}{\sigma^{2}}\right]$$

The fitting procedure was implemented in MATLAB by using global optimization via the GlobalSearch algorithm to identify the values of *k*_LDF_ and σ that minimize Φ. Α more detailed explanation of the MLE fitting algorithm can be found in Low et al.^22^

## 3 Results and Discussion

### 3.1 Textural Properties

Textural properties were measured to quantify the effect of polymerization durations on the pore structure of nonfunctionalized (HCP-1), chlorine-functionalized (HCP–Cl), and DETA-functionalized HCPs (HCP-DETA). The results are presented in Table 1 and Figures 2 and 3. Figures S5 and S6 show the isotherms on a log_10_ scale and are grouped by precursor material to help visualize changes in the isotherm. As seen in Figure 2, and for all three sample sets, the N_2_ isotherms at 77 K show a steep increase at low relative pressures, indicative of the presence of micropores,^54^ while the gradually increasing uptake with relative pressure is indicative of a wide pore size distribution. The desorption branch does not fully close at low relative pressures, which has been observed in other HCPs and is commonly attributed to a flexible pore network^55–57^ and desorption from micropores with restricted access.^58^ The effect of polymerization time on the isotherm shape is readily visible, indicating that differences exist in the resulting textural properties of the synthesized HCPs.Table 1 Figures 2 [Fig fig3].

**Table 1:** Summary of Physical and Chemical Features of the Synthesized HCPs^a^

sample	skeletal density (g/cm^3^)	BET equivalent area (m^2^/g)	*V*_tot_ (cm^3^/g)	*V*_micro_ (cm^3^/g)	*V*_meso_ (cm^3^/g)	*N* content (at %)	Cl content (at %)
HCP-1–10 min	n.d.	1660	1.21	0.56	0.65	n.d.	n.d.
HCP-1–30 min	0.932 ± 0.001	1410	1.35	0.43	0.92	n.d.	n.d.
HCP-1–2 h	1.077 ± 0.002	1320	0.93	0.44	0.49	n.d.	n.d.
HCP-1–19 h	0.888 ± 0.012	1190	0.78	0.42	0.36	nil	2.6
HCP-Cl-10 min	0.860 ± 0.003	1400	1.06	0.46	0.60	n.d.	n.d.
HCP-Cl-30 min	n.d.	1380	1.21	0.42	0.79	n.d.	n.d.
HCP-Cl-2 h	n.d.	1380	0.99	0.46	0.33	n.d.	n.d.
HCP-Cl-19 h	0.900 ± 0.001	1520	1.05	0.55	0.50	nil	4.4
HCP-DETA-10 min	0.882 ± 0.003	670	0.49	0.22	0.27	11.6 (8.89 ± 0.01)	<0.1
HCP-DETA-30 min	0.859 ± 0.003	740	0.75	0.21	0.54	11.1 (9.00 ± 0.09)	<0.1
HCP-DETA-2 h	0.907 ± 0.003	690	0.45	0.23	0.22	11.6 (9.62 ± 0.01)	<0.1
HCP-DETA-19 h	n.d.	370	0.28	0.16	0.12	7.4 (6.31 ± 0.02)	1.2

a Skeletal densities were determined from He pycnometry. The BET areas and micro- and mesopore volumes were determined via N_2_ sorption at 77 K and NLDFT analysis. Nitrogen and chlorine contents were determined via XPS. The N content values in brackets were determined via CHNS elemental analysis. Note: n.d. stands for “not determined” (due to an insufficient amount of sample for accurate measurement).

**Figure 2:**
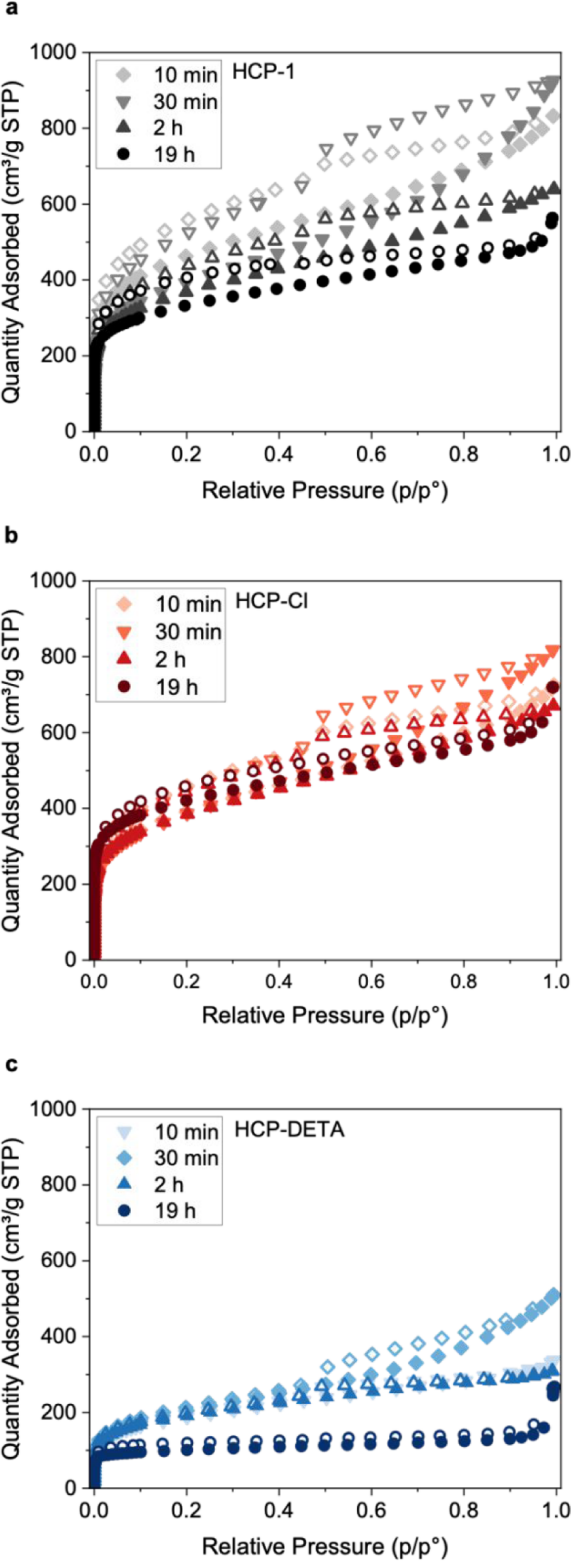
Nitrogen sorption isotherms at 77 K of (a) nonfunctionalized, (b) chlorine-functionalized, and (c) DETA-functionalized HCPs at four different polymerization times. Filled symbols represent adsorption, and hollow symbols represent the desorption branch.

**Figure 3:**
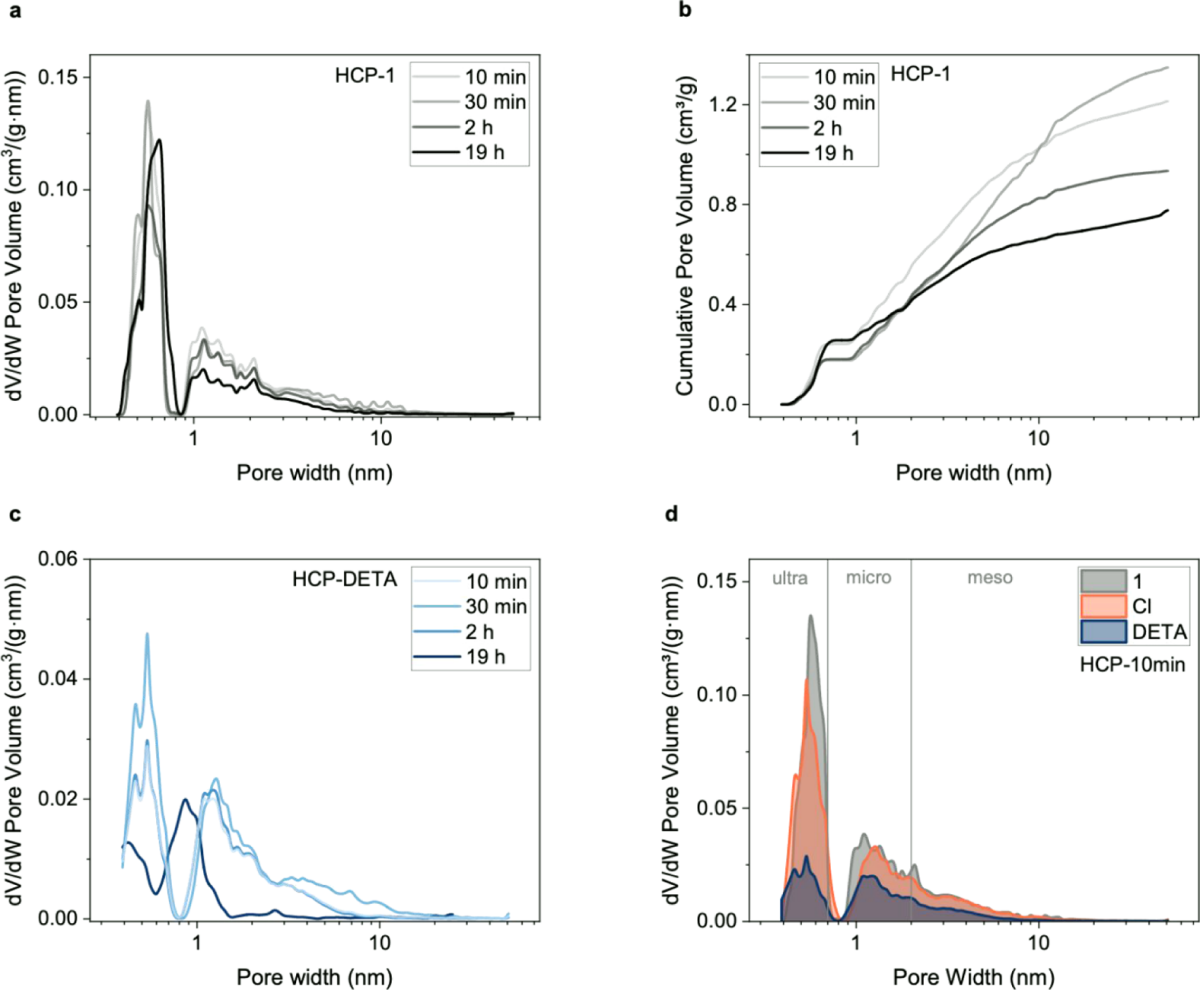
Pore size distribution (PSD) analyses derived from N_2_ sorption at 77 K: (a) differential pore volume of nonfunctionalized polymers (HCP-1 series); (b) cumulative pore volume of the nonfunctionalized HCPs (HCP-1 series); (c) differential pore volume of DETA-functionalized polymers (HCP-DETA series); and (d) differential pore volume of all three HCP samples with a 10 min polymerization step.

Pore size distributions (Figures 3, S8, S9 and S10) were derived from the N_2_ adsorption isotherms at 77 K using 2D-NLDFT, as described in the methods section, with an excellent fit between experimental data and model (Figure S3). Considering the pristine samples (HCP-1 series), longer cross-linking durations tend to reduce the BET area as well as the total pore volume. HCP-1–30 min is a deviation from this trend caused by an increased mesopore volume. The fraction of the micropore volume increases with the polymerization duration, while the fraction of mesopores decreases, whereby HCP-1–30 min also shows a deviation here (Figure S11). We hypothesize that longer polymerization times result in a higher degree of cross-linking and shift the pore size distribution to smaller pore diameter values. This effect would lead to a growing fraction of small pores, some of which may not be detected by the N_2_ sorption analyses at 77 K. The smallest pore diameter which can be detected is around 0.45 nm owing to the kinetic diameter of N_2_ and the pore wall potential. Indeed, the three axes of triptycene form an equilateral triangle of 0.9 nm length if viewed along the *C*_*3*_ symmetry axis with a height of 0.6 nm,^59^ suggesting that cross-linking at multiple sites of the molecule will result in pores smaller than the 0.45 nm “cutoff”. While no detailed studies on the effect of the polymerization duration on the BET area exist for HCPs, previous studies report that polymeric precursors with higher branching extent result in a higher BET area after subsequent Friedel–Crafts cross-linking.^60,61^ This trend appears contrary to our observation and implies that different mechanisms are at play in the two-step HCP synthesis compared to our one-step synthesis.

The chloromethylated HCPs (HCP–Cl series) follow a similar trend as the HCP-1 materials with respect to micropore, mesopore, and total pore volumes (Figure S11). The BET area decreases compared to the nonfunctionalized HCP-1 for short polymerization times, while it increases for the samples with polymerization times of 2 and 19 h. A decrease in surface area was observed by Li et al.^41^ and is explained by the fact that coating of pores with a chloromethyl layer in HCP-Cl reduces the pore surface area and volume. However, a nucleophilic intermediate is formed during the acid-catalyzed chloromethylation, which yields a C–C cross-link as a side product when attacked by an aromatic ring instead of chlorine.^62^ This is a possible route for surface area increase, and it is assumed that the polymers with cross-linking times of 2 and 19 h have a pore size distribution making this side reaction more favorable than the polymers with 10 min and 30 min cross-linking times.

The micro- and mesopore volume of all amine-functionalized samples (HCP-DETA series) is substantially reduced, and the BET area is approximately halved compared to the nonfunctionalized materials (Table 1). A similar change in the BET area is also observed in other studies of chlorine- and amine-tethered HCPs.^42^ A comparison of the pore size distribution at each stage of functionalization shows that the volume contained in small micropores shrinks drastically upon DETA functionalization, while the rest of the micro- and mesopore volumes decrease to a lesser extent (Figures 3d and S10). The DETA molecule has dimensions similar to the peak in the ultramicropore region (i.e., 0.4 × 0.2 × 0.8 nm for the stretched conformer^63^) and therefore likely blocks most of these pores upon functionalization. The ultramicropores remaining after DETA-functionalization either did not react or originated from micropores of diameter around 1 nm, which were grafted with a DETA molecule. The isotherm (N_2_ at 77 K) of HCP-DETA-19 h is flatter than those of the analogues with shorter polymerization duration and does not exhibit a reduction in hysteresis around 0.45 p/p°. This feature suggests a lower mesopore content following the trend observed for the nonfunctionalized precursors (Figures 2c and 3c), which has important implications for the CO_2_ adsorption behavior discussed in Section 2.3.3. Another observation is that the larger pores of the 19 h sample series disappear upon functionalization. This contrasts with the samples resulting from shorter synthesis durations. We hypothesize that the pore morphology of HCP-1–19 h has more constrictions (i.e., narrow pores which connect larger ones), which lead to the effective closure of larger pores (>1.5 nm) upon DETA functionalization. This interpretation is supported by notably higher residual and covalently bonded Cl content in HCP-DETA-19 h compared to other HCP-DETA materials (see Section 3.2 and Figure S16). Indeed, the Cl XPS deconvolution spectrum shows that most chlorine atoms in HCP-1, HCP-Cl, and HCP-DETA are covalently bonded to carbon (C–Cl) and not from residual FeCl_3_ (Figure S16). These observations suggest the larger pores of HCP-DETA-19 h were initially chloromethylated but subsequently inaccessible for DETA substitution. We hypothesize that this situation is due to the high degree of cross-linking in HCP-DETA-19 h. As a result, the sample displays a large fraction of ultramicropores (<0.7 nm diameter) that contain Cl but are inaccessible to DETA. We repeated the synthesis of HCP-1, HCP-Cl, and HCP-DETA for polymerization times of 10 min and 19 h to assess repeatability. The results shown in Figure S7 indicate that there is repeatability in the observed trends, though one sample seems to deviate more (HCP-Cl-19 h).

SEM images were taken to qualitatively inspect morphological changes upon grafting, and representative images are compiled in Figure S14. There are no apparent macroscopic changes in morphology, which is a sign of successful amine grafting. Indeed, aggregation of DETA on the sample surface would indicate additional impregnation of the polyamine.^29,44^ The skeletal density of the hyper-crosslinked polymers is about 0.9 g/cm^3^, and the results are presented in Table 1. Previous studies have reported skeletal densities of other HCPs in the range of 0.9–1.1 g/cm^3^ ^64^ and up to 1.3 g/cm^3^ when employing various cross-linking methods.^65^

### 3.2 Chemical Features

We next analyzed the chemical features of the materials by using FTIR spectroscopy and XPS. FTIR spectra were obtained at all three stages of functionalization of the HCP-19 h polymer to qualitatively compare the presence of functional groups (Figure 4a). The spectra of all compounds contain bands characteristic of the aromatic backbone of HCPs, notably a strong double peak around 1500 cm^–1^ due to aromatic ring modes.^32,66^ Both aromatic C–H stretches (around 3050 cm^–1^) and aliphatic C–H stretches (around 2880 cm^–1^) are observed, confirming the presence of bridging carbon atoms between the benzene rings.^67^ A weak C–Cl peak is observed around 700 cm^–1^ in the chlorine-functionalized HCP that is absent in the other polymers. The amine-functionalized material contains a characteristic C–N stretching peak at 1350 cm^–1^ and a broad peak at 3450–3150 cm^–1^ caused by the N–H stretching modes of primary and secondary amines. Note that the symmetric and antisymmetric stretches are not resolved due to the presence of both primary and secondary amines and the diversity of chemical environments within the amorphous HCP structure.^34^ Overall, the FTIR analysis points to the successful chlorination step, followed by the successful DETA functionalization.

**Figure 4:**
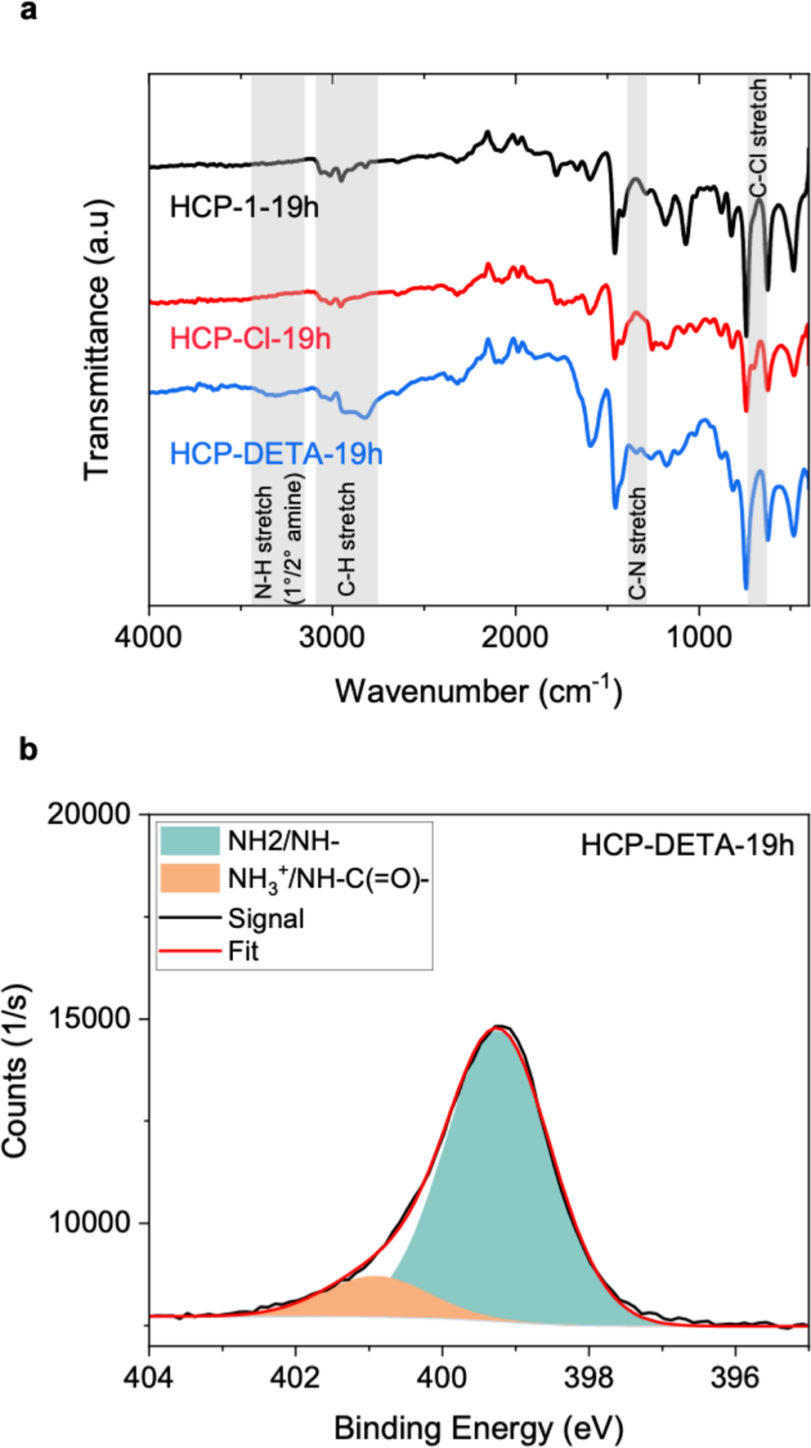
Chemical features characterization of the HCP samples: (a) FTIR spectra of the resulting samples for all three stages of functionalization of HCP-19 h; (b) N 1s deconvolution spectrum of HCP-DETA-19 h.

X-ray photoelectron spectroscopy was carried out to determine the N and Cl contents of the DETA-functionalized HCPs (Table 1, Figures 4b, and S14, S15 and S16). Spectra were also recorded for all intermediates of the 19 h sample to analyze how the chlorine content changes after chloromethylation and DETA-functionalization. As expected, the Cl content increased from 2.6 to 4.4 atom % upon chloromethylation and then decreased to 1.2 at. % upon DETA-functionalization, while the N content increased to 7.4 at. % (Table 1). Using the changes in Cl and N contents during the substitution step, there are on average 1.3 bonds per DETA molecule to the polymer backbone. Assuming mostly primary amines react due to their lower steric hindrance and higher nucleophilicity compared to secondary amines, this estimate results in 23% primary and 77% secondary amines in HCP-DETA-19 h (Note: DETA contains 66% primary amines and 33% secondary amines). A possible origin of Cl in HCP-1 is side reactions during the FeCl_3_-catalyzed cross-linking reaction. Residual iron in HCP-1 was below the detection limit of 0.1 atom %. However, it was shown that HCPs synthesized using FeCl_3_ can contain ppm-level concentrations of iron.^32^

The possible N environments in the sample are primary and secondary amines, carbamic acid or carbamate, and protonated amines (e.g., ammonium carbamate). The N 1s peaks were therefore fitted to two, three, and four Gaussians with 20% Lorentzian mix, respectively. Deconvolution into three and four peaks gave minimal improvement over the fit with two peaks, and due to the relatively large FWHM of the measurement, some of the N environments likely show up as a combined peak in the deconvolution (Figures 4 and S15). The fit with two peaks contains one peak at around 399 eV and a second peak at 400–401 eV, which we assign tentatively to primary + secondary amines and charged amines (carbamate or ammonium), respectively. Another possible origin for the higher-energy peak is urea functional groups, which may be caused by oxidative degradation of the adsorbent.^68^ These binding energies are in line with values reported for amine-functionalized surfaces.^69,70^ The carbamate/ammonium peak would correspond to amine groups that have reacted with CO_2_. Overall, XPS analyses confirm the successful amine-functionalization of the samples whereby samples prepared with the shortest polymerization durations have the highest amine contents.

### 3.3 Equilibrium Adsorption of CO_2_

The equilibrium uptake of CO_2_ was measured at 298 K to assess the suitability of the materials as DAC adsorbents, and the results are presented in Figure 5. The nonfunctionalized and chlorine-functionalized adsorbents are physisorbents and have an almost linear CO_2_ isotherm with little uptake in the low-pressure region. The HCP-1 isotherms show some variation with polymerization time that roughly follows the BET area. Uptake at 1 bar (298 K) is close to 2 mmol/g, which is in line with other nonfunctionalized HCPs reported in the literature.^30,31^ The CO_2_ isotherms of the chlorine-functionalized HCPs are very similar except for HCP-Cl-19 h, which shows higher uptake. We attribute this phenomenon to the increased BET area and ultramicropore volume following chloromethylation of HCP-Cl-19 h (Table 1) and the slightly stronger physisorption of CO_2_ to polar chlorine substituents compared to nonpolar hydrocarbon surfaces. The latter effect and the reduction in BET area may cancel out for the other HCP-Cl samples (i.e., 10 and 30 min) and result in an almost identical uptake compared to their nonfunctionalized precursors. The DETA-functionalized HCPs have high CO_2_ uptakes in the low-pressure region, characteristic of chemisorbents. Shorter polymerization times lead to a higher CO_2_ uptake. Polymers with cross-linking times of 10 min, 30 min, 2 h, and 19 h have CO_2_ uptakes at 400 ppm (and 298 K) of 0.43, 0.32, 0.28, and 0.13 mmol/g, respectively. Part of the explanation for this observation is the higher grafting density of the samples with shorter polymerization time. Indeed, amine-grafted HCPs with polymerization times of 10 min to 2 h have N contents of around 9 mmol/g compared to 6 mmol/g for HCP-DETA-19 h (Table 1). Another part of the explanation might come from the accessibility of the N-containing sites, which we assess below by calculating the amine efficiency. For perspective, Table S3 gives an overview of the CO_2_ uptake of amine-grafted adsorbents of other material classes. To assess repeatability, we also measured the CO_2_ uptake of the samples whose synthesis was repeated. The results reported in Figure S7 show that the CO_2_ uptakes of HCP-DETA-10 min and HCP-DETA-19 h at 400 ppm vary by 1% to 30% between the first and second synthesis and by about 10% at 1 bar.

**Figure 5:**
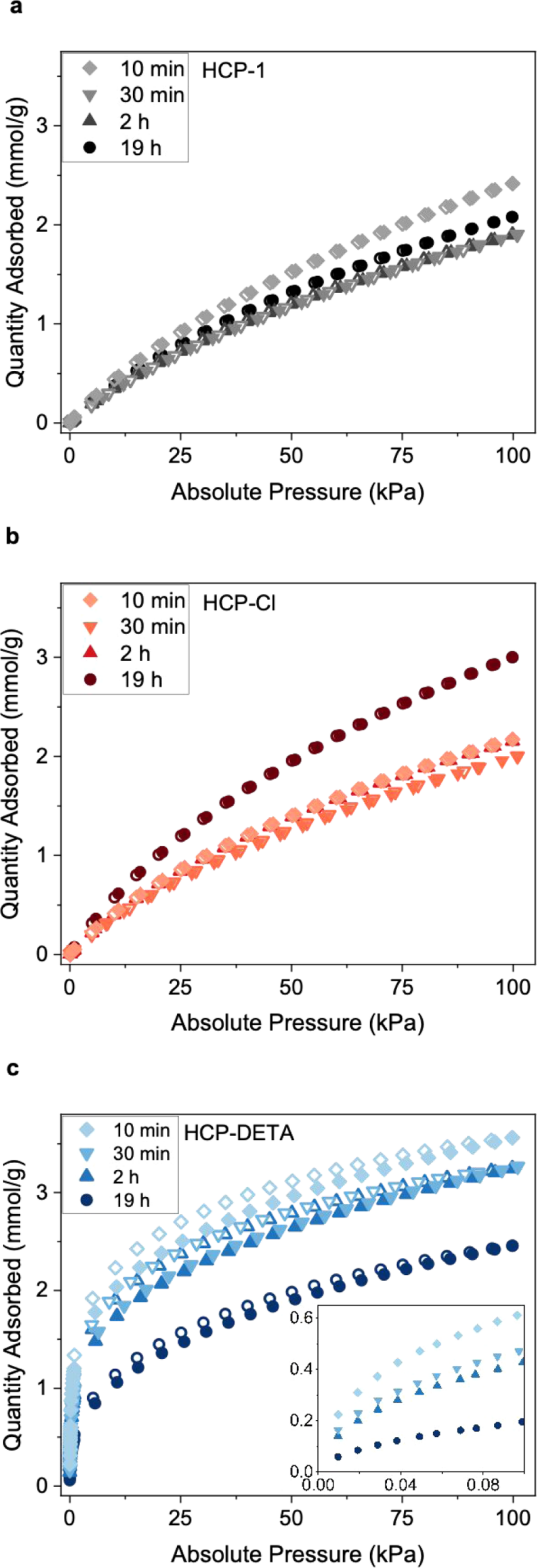
CO_2_ sorption isotherms at 298 K for (a) nonfunctionalized HCPs, (b) chlorine-functionalized HCPs, and (c) DETA-functionalized HCPs at four different polymerization times. The inset in (c) shows the low-pressure regions to highlight uptake at 400 ppm (i.e., 0.04 kPa).

The amine efficiencies at 400 ppm are 9%, 7%, 6%, and 4% for HCP-DETA-10 min, HCP-DETA-30 min, HCP-DETA-2 h, and HCP-DETA-19 h (Table S2) and were calculated using the equation below

$$\eta \left(P\right)=\frac{2q\left(P\right)}{\sigma }$$

where η­(*P*) is the amine efficiency at pressure *P*, *q*(*P*) is the adsorbed amount of CO_2_ at pressure *P* (in mmol/g), and σ is the amine density of the material (in mmol/g). First, these values suggest that most of the N sites are not interacting with CO_2_. This may be caused by DETA molecules grafted into micropores that are not accessible to CO_2_. For instance, amine-functionalized adsorbents with larger pores, such as Lewatit, achieve amine efficiencies in the region of 25% under comparable conditions. Besides, the trend suggests that shorter polymerization time leads to increased amine efficiency. It is possible that this trend continues outside of the investigated range, such that materials with shorter polymerization times than 10 min may achieve higher amine efficiencies. However, going below a polymerization time of 10 min might make the synthesis difficult to control due to the comparatively short heating duration. Such practical issues of reducing the polymerization time even further may be circumvented by choosing lower reaction temperatures to more precisely control the degree of polymerization or by using a microfluidic reactor for the synthesis. The accessibility of the amine sites is also affected by the porosity of the samples, as indicated by the different pore size distributions of the four HCP-DETA adsorbents. The observed amine efficiencies at atmospheric CO_2_ concentrations between 4% and 9% are of the same order of magnitude as the 10% of amine groups bonding CO_2_ observed using XPS.

Characteristic of polymeric adsorbents with low surface charge densities (i.e., van der Waals interactions are dominating), HCPs have typically low nitrogen uptakes at ambient temperatures. This is an advantage over ion-containing materials such as MOFs and zeolites, which adsorb more N_2_ at ambient conditions due to electrostatic interactions with the quadrupole of the nitrogen molecule.^71^ Nitrogen isotherms were obtained for HCP-DETA and all derivatives of HCP-19 h, and uptakes at 298 K and 100 kPa of around 0.1 mmol/g were observed for all HCP-DETA samples (Figure S12). Linear isotherms characteristic of physisorption were observed, similar to other amine-functionalized HCPs.^20,28,36,38,42^ In summary, all amine-functionalized adsorbents show chemisorbing properties and low nitrogen adsorption, allowing for the highly selective adsorption of CO_2_ from the atmosphere. HCP-DETA-10 min is the best-performing adsorbent with an uptake of 0.43 mmol/g (at 298 K and 0.04 kPa), which is the highest reported value for an amine-grafted HCP and about half of the benchmark adsorbent Lewatit VP OC 1065.^20^

### 3.4 Equilibrium Sorption of H_2_O and Effects of H_2_O on HCPs

Water sorption isotherms were recorded for DETA-functionalized adsorbents. All four samples show increasing mass uptake as relative humidity increases, which is characteristic of hygroscopic materials (Figure 6). However, the extent and rate of uptake differ significantly between the samples, indicating differences in their affinity for water vapor and possibly their pore structure, chemistry, or surface functionality. HCP-DETA-19 h has the lowest water uptake due to its lower porosity and lower amine content. The other HCP-DETA materials show a similar water uptake behavior, including a rapid increase in water adsorption at high partial pressures, which is indicative of capillary condensation.

**Figure 6:**
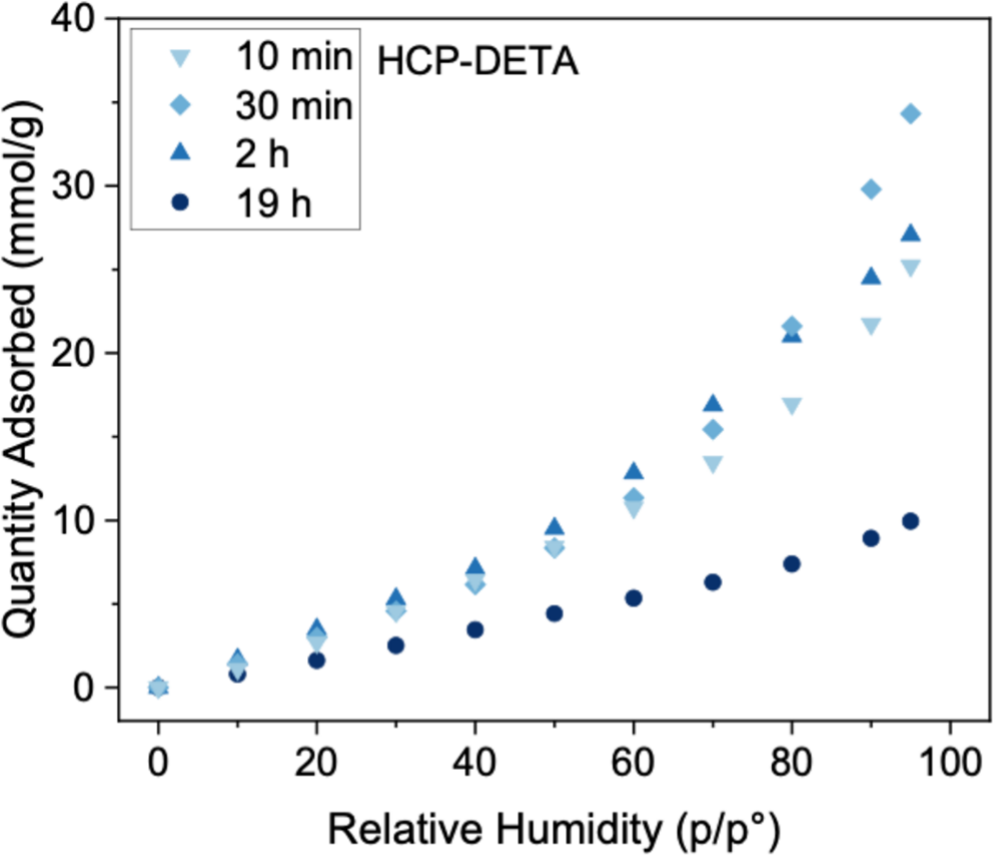
Water adsorption isotherms of HCP-DETA samples at 298 K.

Water coadsorption was not investigated; however, in line with other amine-functionalized adsorbents, we expect there to be a favorable impact on the equilibrium CO_2_ adsorption and adsorption kinetics.^72^ For instance, the presence of water increases equilibrium CO_2_ uptake at 400 ppm by 60 to 90%^73^ in the case of Lewatit and 110% for COF-999 ^74^ at 30 and 50% RH, respectively. No significant swelling was observed in 50% and 100% relative humidity at 298 K (Figure S23). Although not determined in this study, it is likely that the presence of humidity will alter the optimum cross-linking time for highest CO_2_ uptake performance.

### 3.5 CO_2_ Adsorption Kinetics

Adsorption kinetics are an important factor contributing to the overall process cost, and hence, are required to assess the suitability of a DAC adsorbent. We selected the samples with the largest and lowest equilibrium capacity at 400 ppm CO_2_ (HCP-DETA-10 min and HCP-DETA-19 h) to carry out kinetic measurements via thermogravimetric analysis (TGA). Additionally, we measured the adsorption kinetics of the benchmark Lewatit VP OC 1065 using particles of the same size range as the HCPs (24–75 μm) to allow for comparison. The results are summarized in Figure 7 and show that both HCPs exhibit significantly faster uptake than Lewatit. The LDF constant of the CO_2_ uptake on HCP-DETA-10 min at 400 ppm was determined to be 0.0120 ± 0.0004 s^–1^. The parameter uncertainty of the MLE fit is given by the covariance matrix and confidence ellipse in Figure S17. As expected from the smaller average pore size of HCP-DETA-19 h, the kinetic constant is reduced to 0.0092 ± 0.0001 s^–1^ due to more diffusional resistance (Figures 7a and S19). However, these two values remain very close in comparison to Lewatit, showing that kinetics are not affected as much as uptake. Indeed, the two samples are approximately 4 to 6 times faster than the benchmark adsorbent Lewatit, which has an LDF constant of 2.167 ± 0.006 × 10^–3^ s^–1^ (Figure S21).

**Figure 7:**
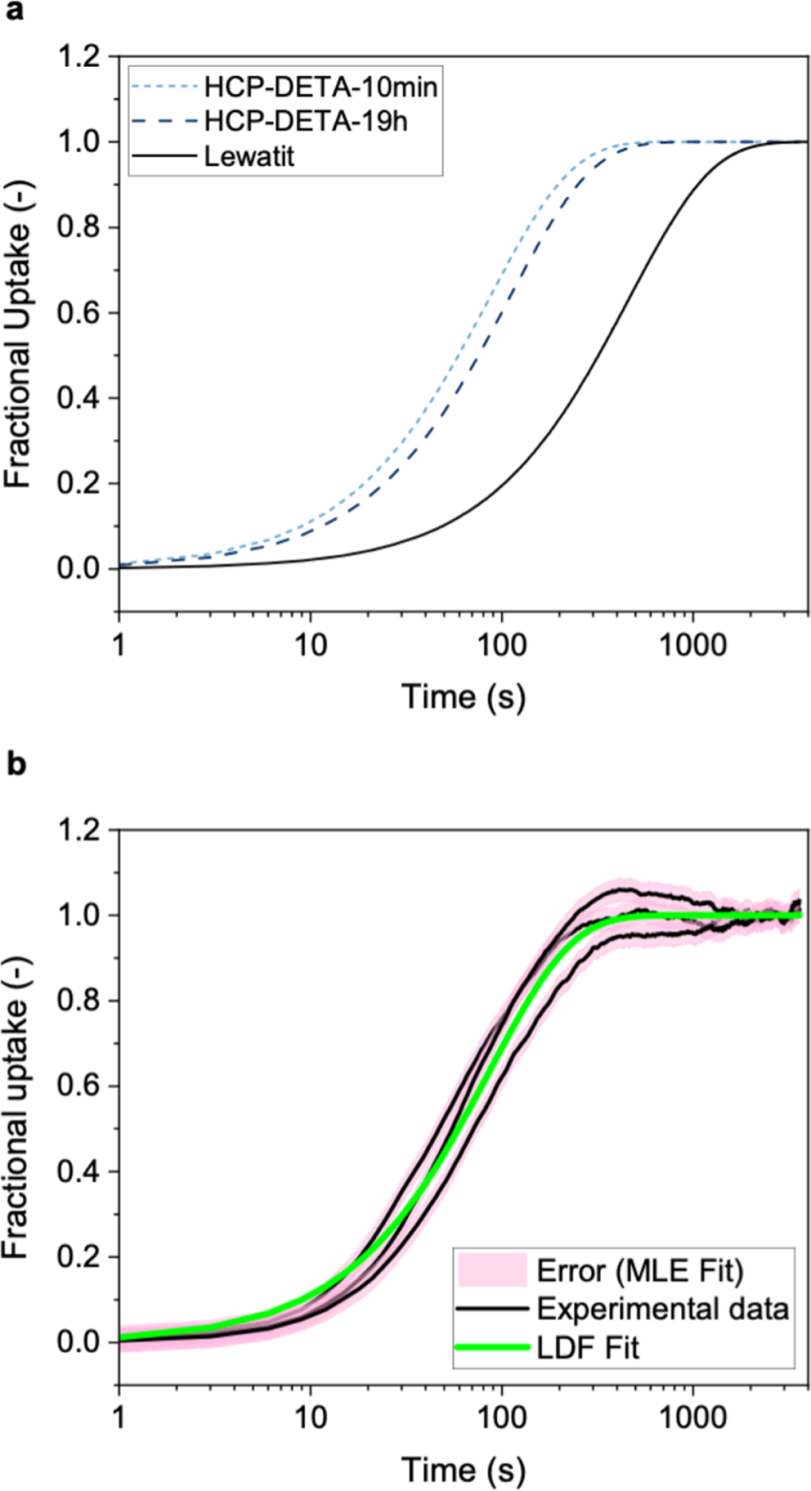
CO_2_ sorption kinetics analyses under 400 ppm of CO_2_ in He at a flow rate of 400 mL/min at 303 K: (a) adsorption kinetics of HCP-DETA-10 min and HCP-DETA-19 h compared to Lewatit VP OC 1065 (all 24–74 μm particle size fraction) determined by fitting the TGA uptake measurement using the LDF model; (b) Fractional uptake curves (three independent runs) of HCP-DETA-10 min together with the estimated experimental error and LDF fit. Three repeat measurements were carried out for each material, and the LDF fit was obtained using the MLE treatment (Figure S21).

To ensure that the observed mass transfer kinetics are those of the sample and not due to insufficient CO_2_ flow to the sample, a flow rate study was conducted. Uptake curves using 300, 350, and 400 mL/min of 400 ppm of CO_2_ in He were obtained and fitted to the LDF model (Figures S18 and S19). Measurements at each flow rate were carried out three times and fit using maximum likelihood estimation (MLE). TGA uptake curves for measurements at 300 and 350 mL/min and the MLE confidence ellipses are given in Figure S19. Overall, within the flow rate range studied, there was no clear change in the kinetics, which suggested that external mass transfer limitations due to the setup were not significant (Figure S20). The reported k_LDF_ value of HCP-DETA-10 min was determined at a flow rate of 400 mL/min, showing the smallest standard deviation out of all flow rates (Figures S18 and S19). The match between the LDF fit and a single uptake curve was observed to improve after approximately 25 s following exposure to 400 ppm of CO_2_ (Figure S21). Measurements of the CO_2_ concentration at the outlet of the TGA furnace indicate that 90% of the equilibrium atmosphere is established within this time frame (Figure S21a–c). This suggests that the slower initial uptake, compared to the LDF prediction, is due to the gradual establishment of the CO_2_ atmosphere rather than an instantaneous step change from 0 to 400 ppm. The LDF constant obtained by fitting the CO_2_ concentration within the TGA (i.e., light blue curves in Figure S20a–c) is six times greater than the LDF constant of the CO_2_ uptake on HCP-DETA-10 min. In other words, the response time of the equipment is six times faster than the response time relating to the samples’ kinetics. While this difference is needed, it is not as much as we would hope for a fully quantitative measurement.^75^ For this reason, it must be assumed that the stated LDF constant is rather a lower bound than a definite value.

The kinetics of amine-based adsorbents are mainly affected by (1) the diffusion of CO_2_ within the pores of the material and (2) the rate of reaction (i.e., chemisorption). Determining the reaction rate of a single amine layer with CO_2_ at 400 ppm remains a challenge which is of great interest to the DAC adsorption development, as this would provide a chemisorption rate constant that can be used to deconvolute reaction and diffusion barriers. While no measurements of the CO_2_ adsorption kinetics on a single amine layer exist (i.e., the rate of the chemisorption reaction), measurements of the reaction rates of polyethylenimine (PEI) films of various thicknesses with 400 ppm of CO_2_ using a quartz crystal microbalance can provide some perspective, though PEI and DETA have different proportions of primary amines.^76^ In this work, Hoffman et al. report *k*_LDF_ values of 0.0078 s^–1^ and 0.0196 s^–1^ for the 10 nm PEI films at 298 and 308 K, respectively. These values are on the same order of magnitude as the lower bound value for HCP-DETA-10 min of 0.0120 ± 0.0004 s^–1^ at 303 K and may indicate that amine–layer reaction barriers are rate limiting, instead of diffusional resistances, in the HCP particles investigated in our study. However, this remains a qualitative comparison, as the relationship between chemical and diffusional reaction barriers in the HCP is complex, and the adsorption kinetics on the 10 nm PEI film not only describe the chemical barrier at the surface but also diffusion through the film. In another study where the adsorption kinetics of Lewatit particles of different sizes were determined, Bos et al. found that adsorption kinetics are improving with smaller particle size. However, no further increase in kinetics is achieved for particles smaller than 150–250 μm (at 100 mbar, 313 K), which the authors attribute to the isolated amine reaction rate constant. When the uptake curve (at 100 mbar, 303 K) of these particles is fit to a PSO model, a reaction constant of 0.032 s^–1^ is obtained, which is comparable to the k_LDF_ measured in this study.^77^

In contrast, commercially available Lewatit VP OC 1065 (beads with diameters ranging from 300 to 1200 μm) has an LDF rate constant of 3.1 × 10^–4^ s^–1^ at 303 K and 400 ppm.^22^ This is about 40 times slower than the lower bound measured for HCP-DETA-10 min. This trend is most likely due to the larger particle size of Lewatit beads compared to the HCP particles (25–74 μm). Indeed, the 25–74 μm particle size Lewatit sample we measured here exhibited 7 times faster kinetics compared to the 300 to 1200 μm beads of the same material. These findings emphasize that amine-functionalized polymers are not entirely controlled by the chemisorption reaction barrier but that macro- and mesopore diffusion also play a part, as has been indicated by other studies.^78–81^ This observation calls for systematic pore structure engineering to enhance reaction kinetics while keeping the chemistry and equilibrium CO_2_ uptake the same. Adsorbents which have a tunable pore structure over a wide range (e.g., micro- to macropores) would be of particular interest here, as a change in diffusion kinetics will be more apparent compared to the micro- and mesoporous materials covered in this study.

## 4 Conclusion

This study examined the influence of the degree of polymerization on the porosity and CO_2_ uptake of amine-functionalized hyper-crosslinked polymers under DAC conditions. The results reveal that increased crosslinking duration leads to reduced pore volume, particularly in the larger micropore and mesopore ranges. We link this trend to the extensive crosslinking, which shifts a substantial fraction of the pore volume into sizes smaller than the kinetic diameter of N_2_, rendering them undetectable as accessible pore space. The increase in pore volume at shorter crosslinking times resulted in higher CO_2_ uptake and amine efficiency of the DETA-grafted materials. The polymer prepared with the shortest crosslinking time, HCP-DETA-10 min, exhibited the highest CO_2_ uptake of 0.43 mmol/g at 400 ppm of CO_2_ (298 K), outperforming materials prepared at 30 min (0.32 mmol/g), 2 h (0.28 mmol/g), and 19 h (0.13 mmol/g). Since amine grafting densities were largely consistent across the samples, the superior performance of HCP-DETA-10 min can be attributed to its enhanced amine accessibility caused by its pore structure. Kinetic analysis of HCP-DETA-10 min using an LDF model fit to TGA uptake curves at 400 ppm of CO_2_ revealed a lower bound k_LDF_ value of 0.0120 ± 0.0004 s^–1^ for particles in the 24–74 μm range. This value is 40 times higher than that of the larger (0.3–1.2 mm) beads of the benchmark amine-functionalized adsorbent Lewatit VP OC 1065, indicating that internal diffusion limitations, coupled with chemisorption barriers, govern adsorption kinetics. These findings highlight the importance of controlling crosslinking duration to optimize polymer porosity, equilibrium CO_2_ adsorption at 400 ppm, and amine efficiency in HCPs. They open avenues for future research into tailoring the support structures of amine-functionalized porous polymers to achieve optimal CO_2_ capture performance. Especially increasing the pore volume of larger micro- and mesopores is expected to result in a higher amine efficiency and equilibrium uptake. Studies of water coadsorption and stability under realistic process conditions will be essential to further evaluate and enhance the practical applicability of these materials.

## Supplementary Material



## Notes

Raw experimental data of the following characterization techniques is available in a compressed zip file: textural characterization of the materials (AIF), kinetic measurements via TGA, FTIR, XPS, and SEM (PDF)
