# Supplementary material for: Amine-Appended Hyper-Crosslinked Polymers for Direct Air Capture of CO2
Source: ACS Sustain Chem Eng. 2026 Jan 17;14(4):1834–46. doi: 10.1021/acssuschemeng.5c08715 (PMC12869486; doi:10.1021/acssuschemeng.5c08715)
Supplement: Supplementary file 1 [file sc5c08715_si_001.pdf]

Supporting Information

Amine-appended hyper-crosslinked polymers for direct air capture of CO_2

Tristan L. Spreng[1], David Danaci[1,2], Preshti D. Ram[1], Daryl R. Williams[1], Ronny Pini[1,2], Camille Petit[1]*

[1]Department of Chemical Engineering, Imperial College London, London SW7 2AZ, United Kingdom

[2]The Sargent Centre for Process Systems Engineering, Imperial College London, London SW7 2AZ, United Kingdom

[3]I-X Centre for AI in Science, Imperial College London, London W12 0BZ, United Kingdom

*Corresponding author: camille.petit@imperial.ac.uk

Table of Contents

Figure S1: Amine functional groups 3

Figure S2: Hyper-crosslinked polymer (HCP) synthesis scheme 4

Figure S3: Model fit of 2D-NLDFT analysis 5

Figure S4: TGA uptake measurement error correction 6

Figure S5: Nitrogen adsorption isotherms at 77 K of HCP-1, HCP-Cl, and HCP-DETA 7

Figure S6: Nitrogen adsorption isotherms at 77 K of HCP-10min, -30min, -2h, and -19h 8

Figure S7: Adsorption isotherms of re-synthesised HCPs (N_2 at 77 K and CO_2 at 298 K) 9

Figure S8: Pore size distribution (PSD) of HCP-Cl 10

Figure S9: Cumulative pore volume of HCP-Cl and HCP-DETA 11

Figure S10: PSDs of HCP-30min, -2h, and -19h 12

Figure S11: Pore volume change analysis …… 13

Figure S12: Nitrogen adsorption isotherms at 298 K of HCP-19h and HCP-DETA 14

Figure S13: Scanning electron microscopy (SEM) images of HCP-19h 15

Figure S14: X-ray photoelectron spectroscopy (XPS) spectra of HCP-19h and HCP-DETA 16

Figure S15: Deconvolution of N 1s XPS peaks of HCP-DETA 17

Figure S16: Deconvolution of Cl 2p XPS peaks of HCP-19h 18

Figure S17: Statistical analysis of TGA uptake measurements at 400 mL/min flow rate 19

Figure S18: TGA uptake measurement and analysis at 300 and 350 mL/min flow rates 20

Figure S19: TGA flow rate study 21

Figure S20: TGA uptake and CO_2 concentration kinetics 22

Figure S21: TGA uptake and analysis of HCP-DETA-19h and Lewatit VP OC 1065 23

Figure S22: Swelling experiments of HCP-DETA-19h 24

Table S1: XPS fitting parameters for N 1s peak deconvolution 25

Table S2: Amine content and efficiency of HCP-DETA 26

Table S3: Comparison of HCP-DETA-10min to other amine-grafted sorbents 27

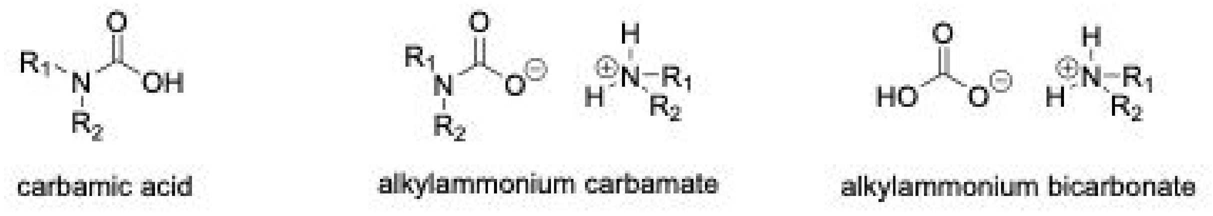

Figure S1. Chemical species forming after the nucleophilic attack of an amine group onto carbon dioxide.

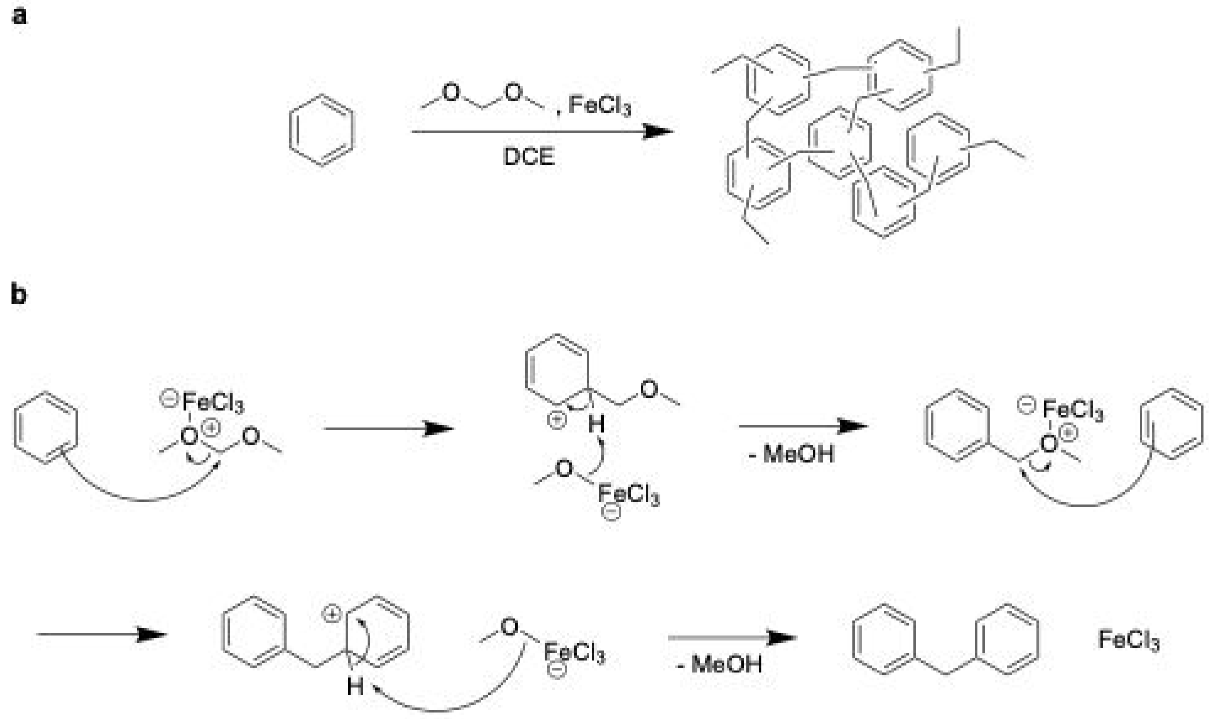

Figure S2: a) General synthesis scheme and b) reaction mechanism for the formation of hyper-crosslinked polymers. The benzene monomer is representative of the aromatic units in triptycene.

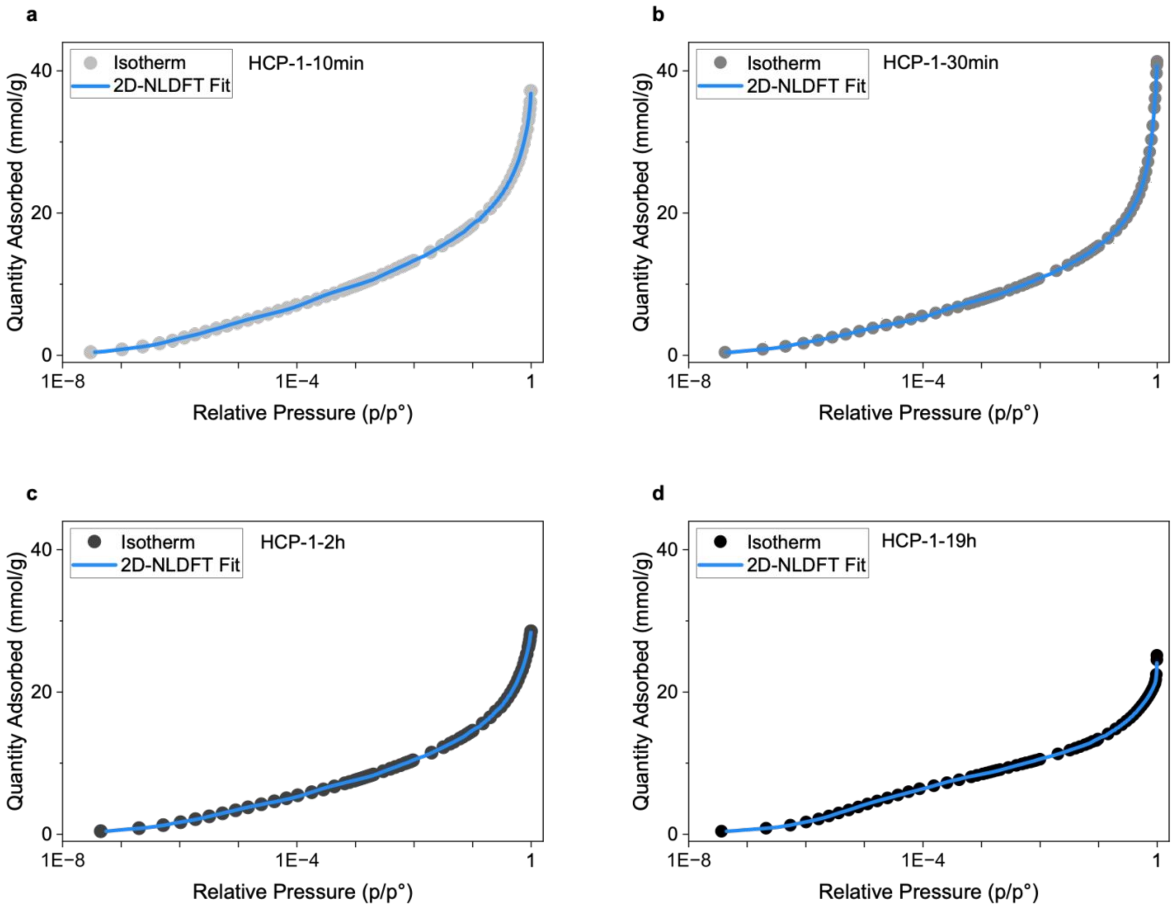

Figure S3: Fit of 2D-NLDFT model to N_2 77K adsorption isotherm of HCP-1 with: a) 10 min, b) 30 min, c) 2 h and d) 19 h polymerisation times.

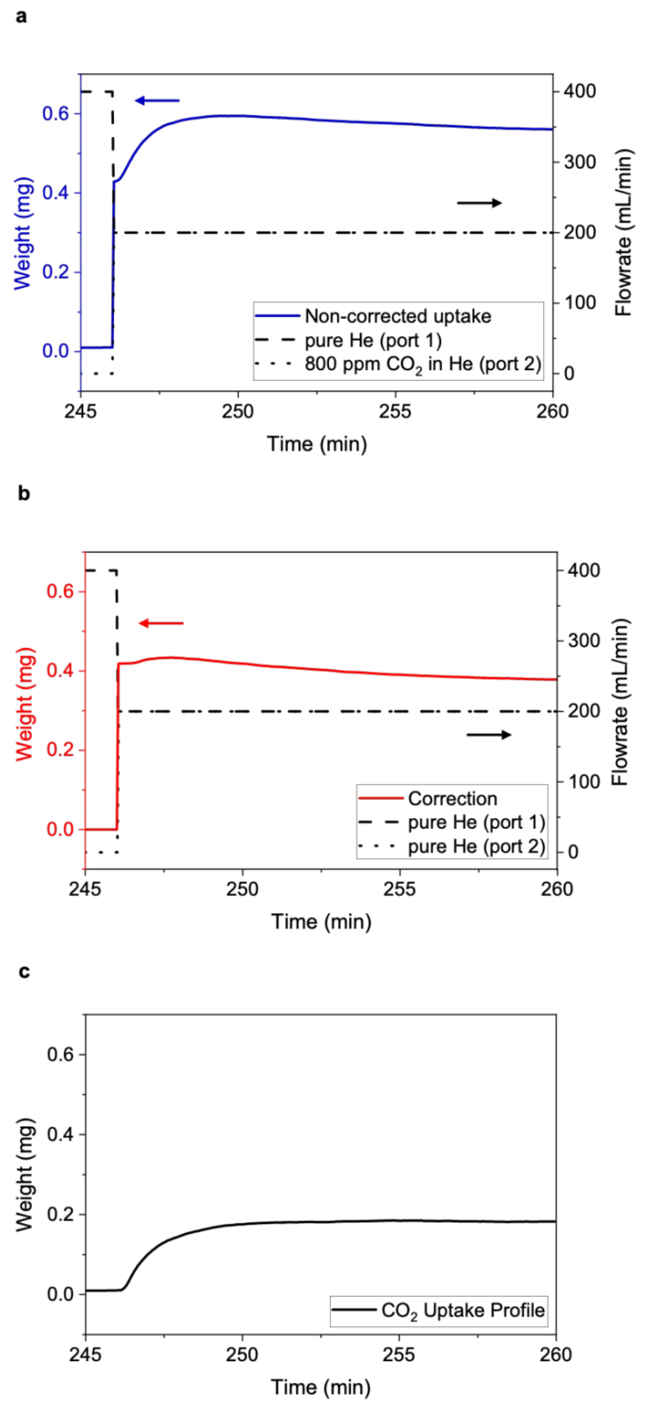

Figure S4: CO_2 sorption kinetics measurement of HCP-DETA-10min with 400 mL/min flowrate. This figure shows how the correction file is applied to the raw TGA data to obtain the correct mass profile. Changes in buoyancy occur within the first minute of switching the gases and have an identical magnitude for both the measurement and the correction file. The CO_2 uptake kinetics occur on timescales of several minutes and are separated from the buoyancy effects in the corrected file.

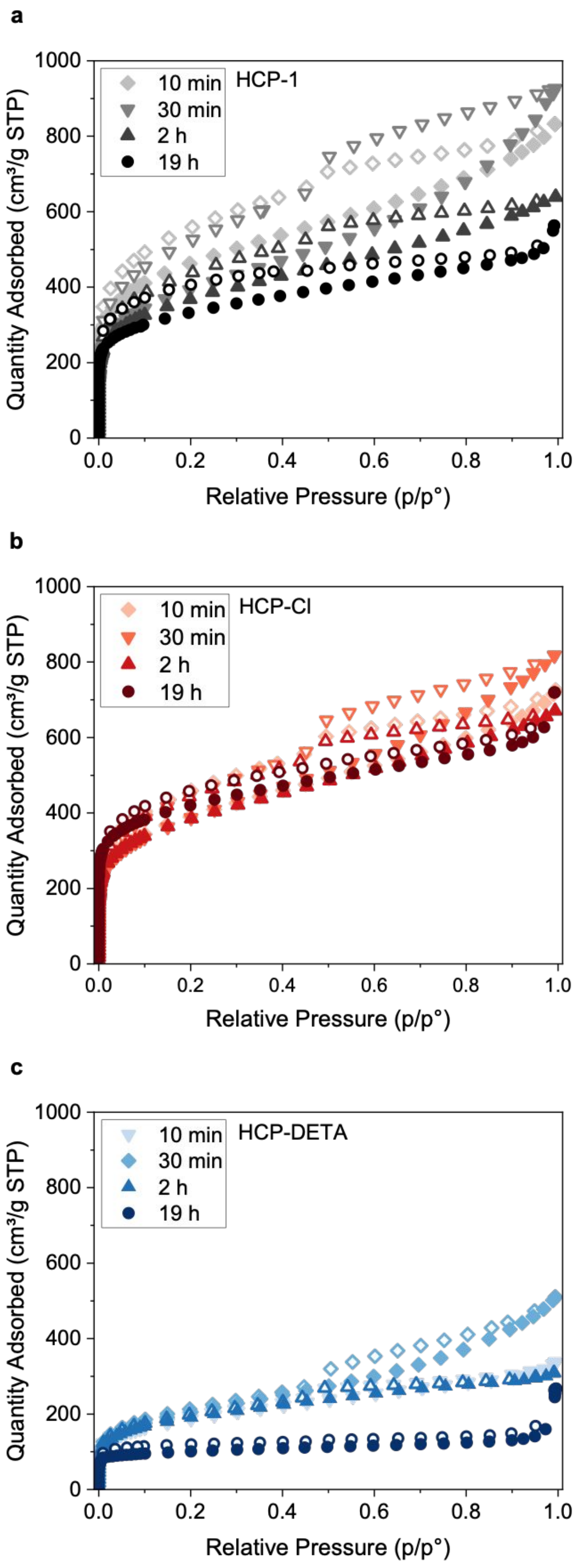

Figure S5: Nitrogen adsorption isotherms at 77 K of a) HCP-1, b) HCP-Cl and c) HCP-DETA plotted on a log10 axis.

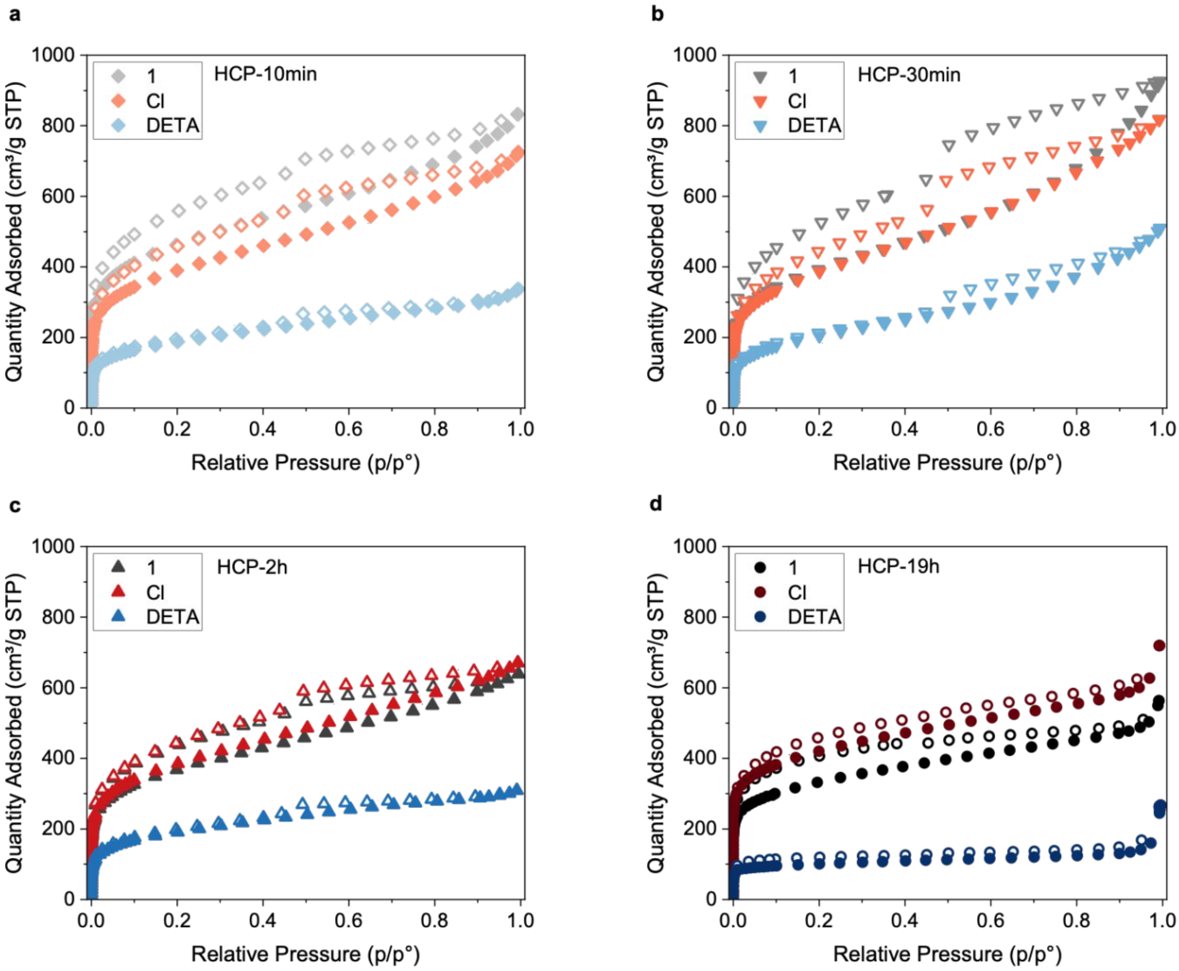

Figure S6: Nitrogen adsorption isotherms at 77 K of a) HCP-10min, b) HCP-30min, c) HCP-2h and d) HCP-19h. Materials synthesised from the same precursor are grouped in the same plot to visualize how the isotherm changes with different types of functionalisation.

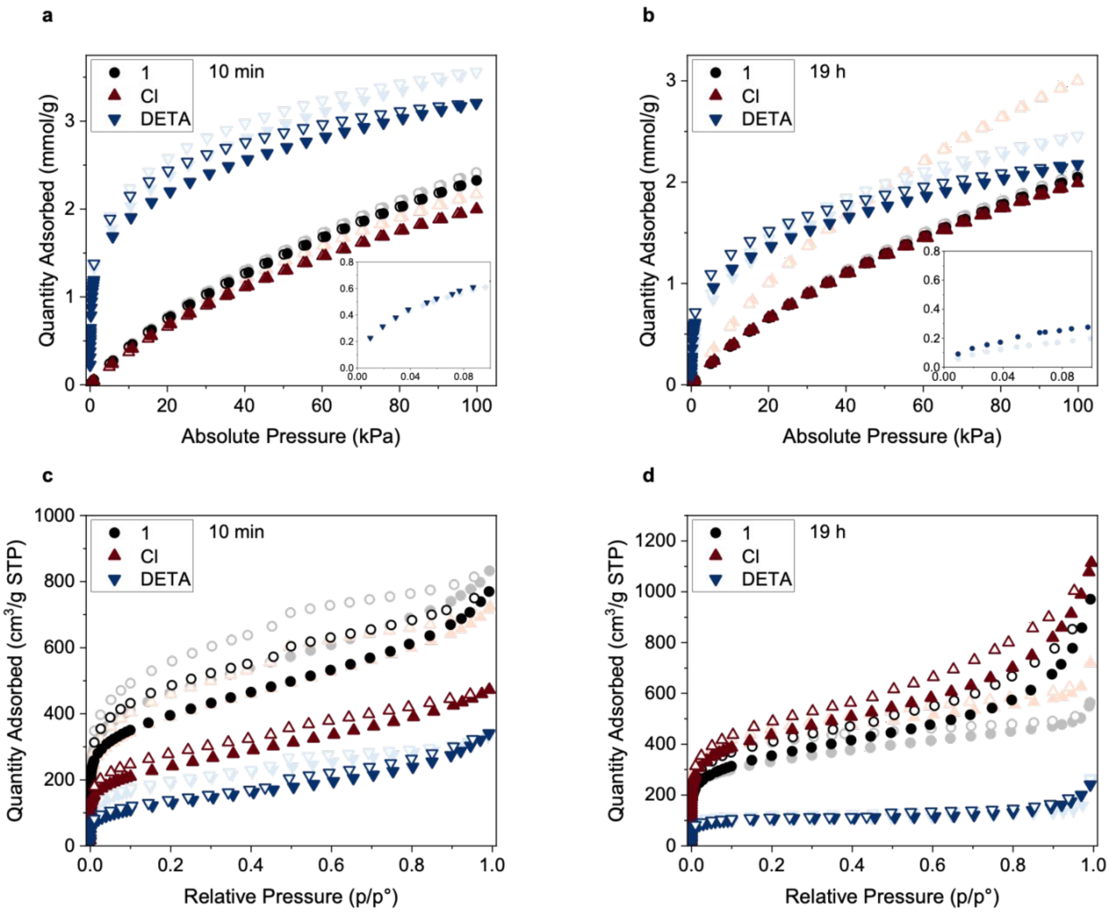

Figure S7: Repeat isotherm measurements with re-synthesised HCP-10min and HCP-19h. CO_2 isotherms at 298 K of a) HCP-DETA-10min and b) HCP-DETA-19h. Nitrogen isotherm at 77 K of c) HCP-DETA-10min and d) HCP-DETA-19h. Triptycene was sourced from TCI in the first synthesis run (light colors) and from Sigma Aldrich in the re-synthesis run (dark colors).

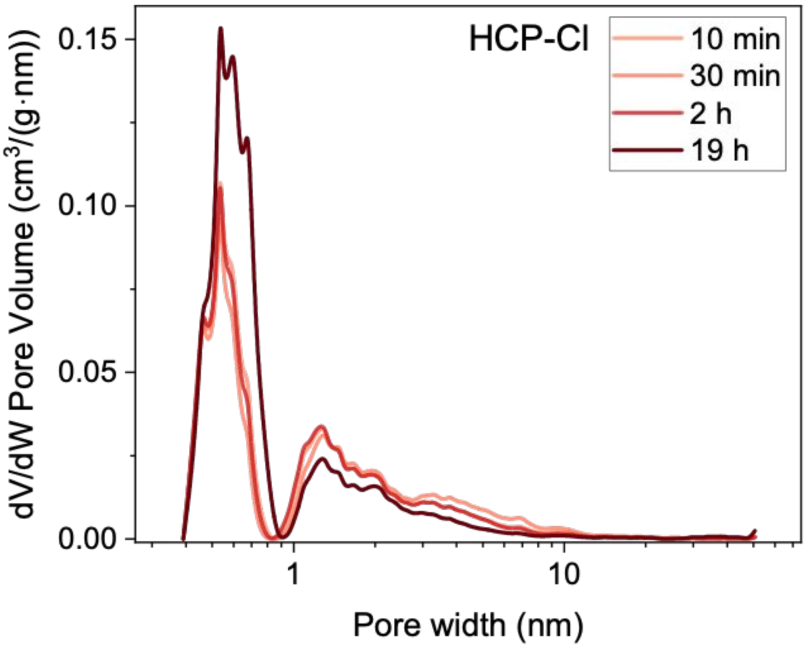

Figure S8: Comparison of pore size distributions of HCP-Cl as derived from N_2 sorption at 77 K and using the 2D-NLDFT model.

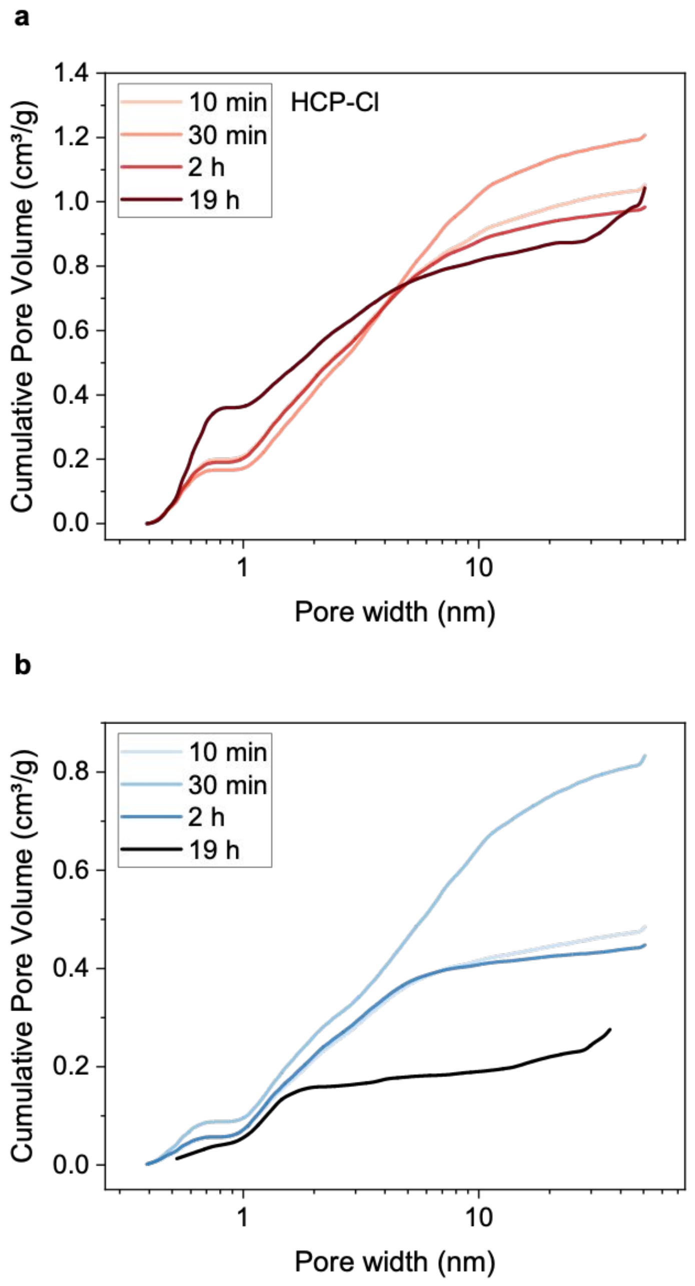

Figure S9: Cumulative pore volume of a) HCP-Cl and b) HCP-DETA as derived from N_2 sorption at 77 K and using the 2D-NLDFT model.

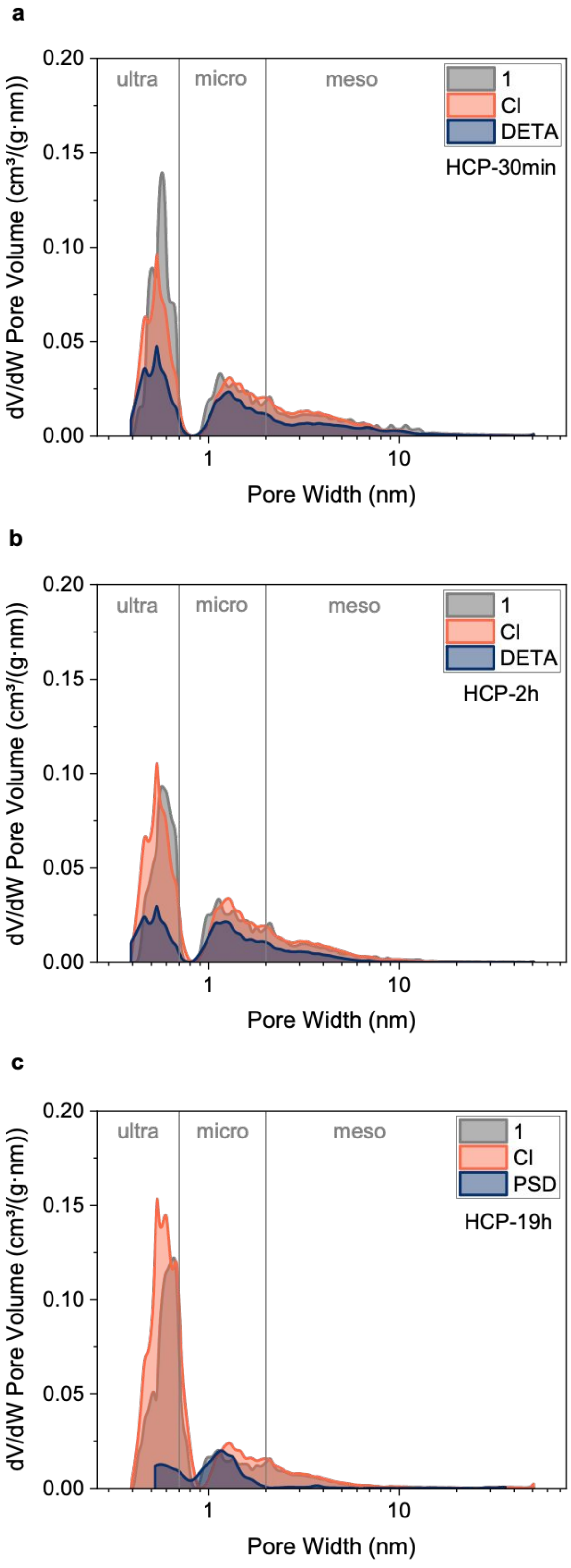

Figure S10: Comparison of the pore size distributions derived from N_2 sorption measurements at 77 K for different functionalization stages for: a) HCP-30min, b) HCP-2h and c) HCP-19h.

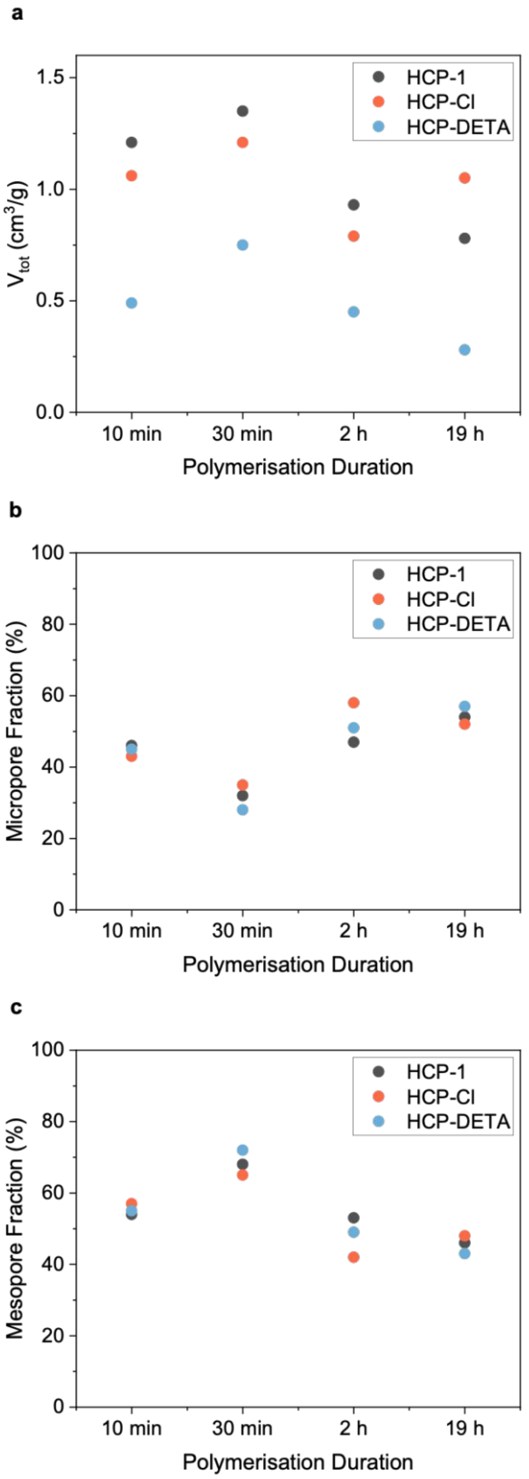

Fig. S11: Summary of pore volume changes in the micro- to mesopore range for polymers of various crosslinking durations and functionalisation types, as derived from N_2 sorption measurements at 77 K. a) Total pore volume (i.e. sum of micro- and mesopore volume, b) fraction of micropore volume of the total pore volume and c) fraction of mesopore volume of the total pore volume.

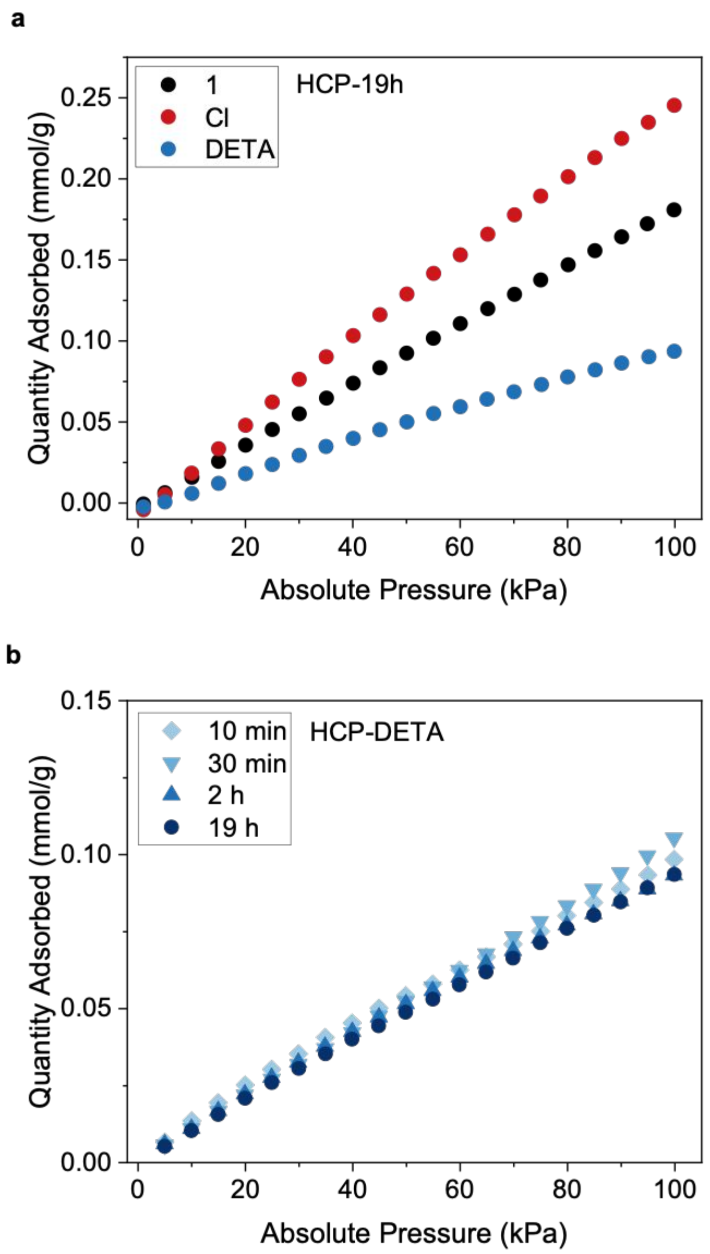

Figure S12: Nitrogen adsorption isotherms at 298 K of a) HCP-19h and b) HCP-DETA.

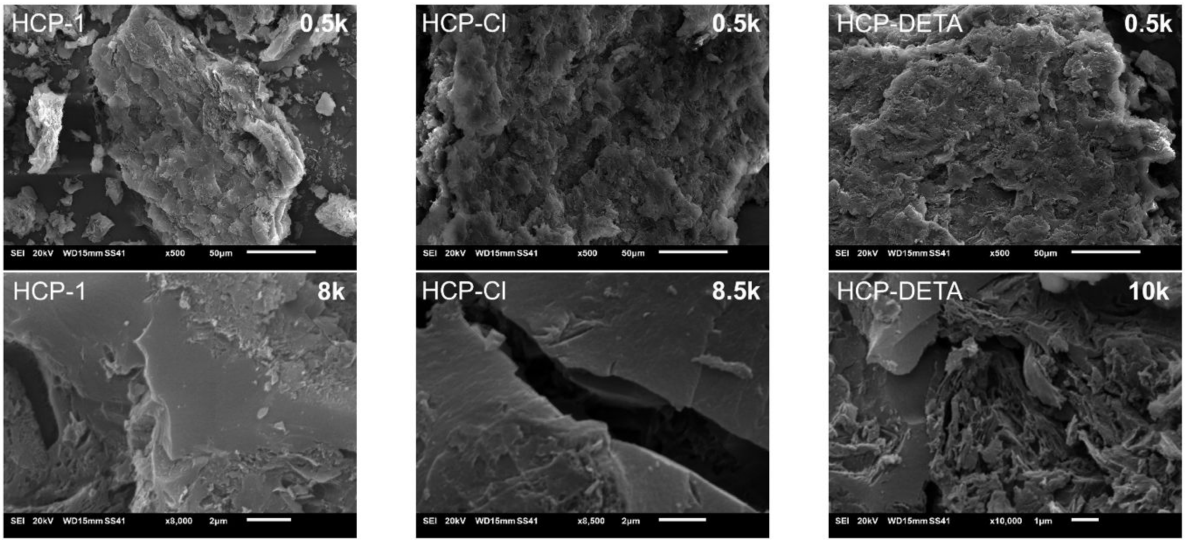

Figure S13: Scanning electron microscope images of representative regions of HCP-1-19h (first column), HCP-Cl-19h (second column), and HCP-DETA-19h (third column).

a

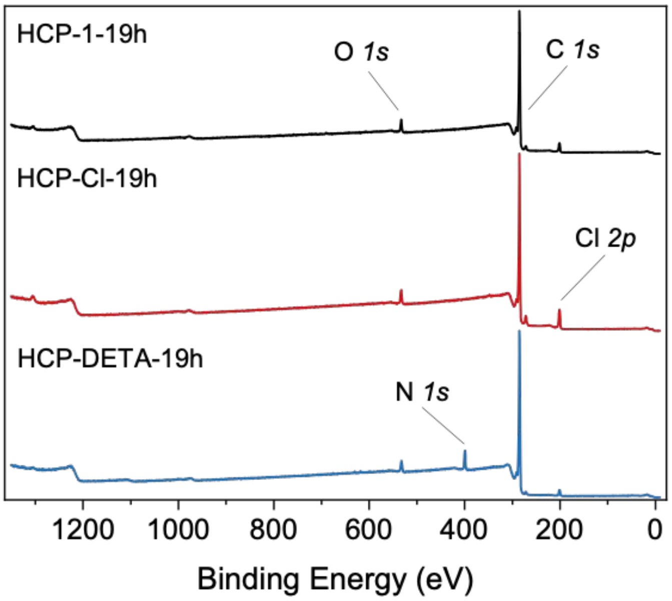

b

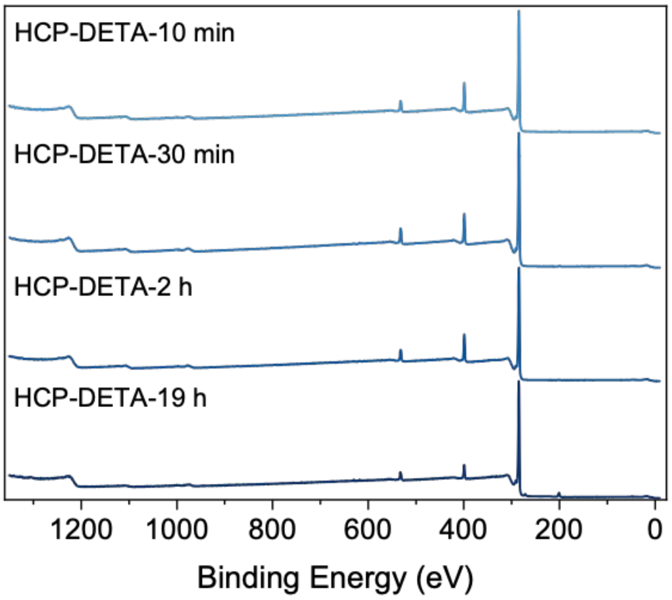

Figure S14: X-ray photoelectron survey spectra of a) all adsorbents resulting from a 19h polymerisation step and b) all HCP-DETA.

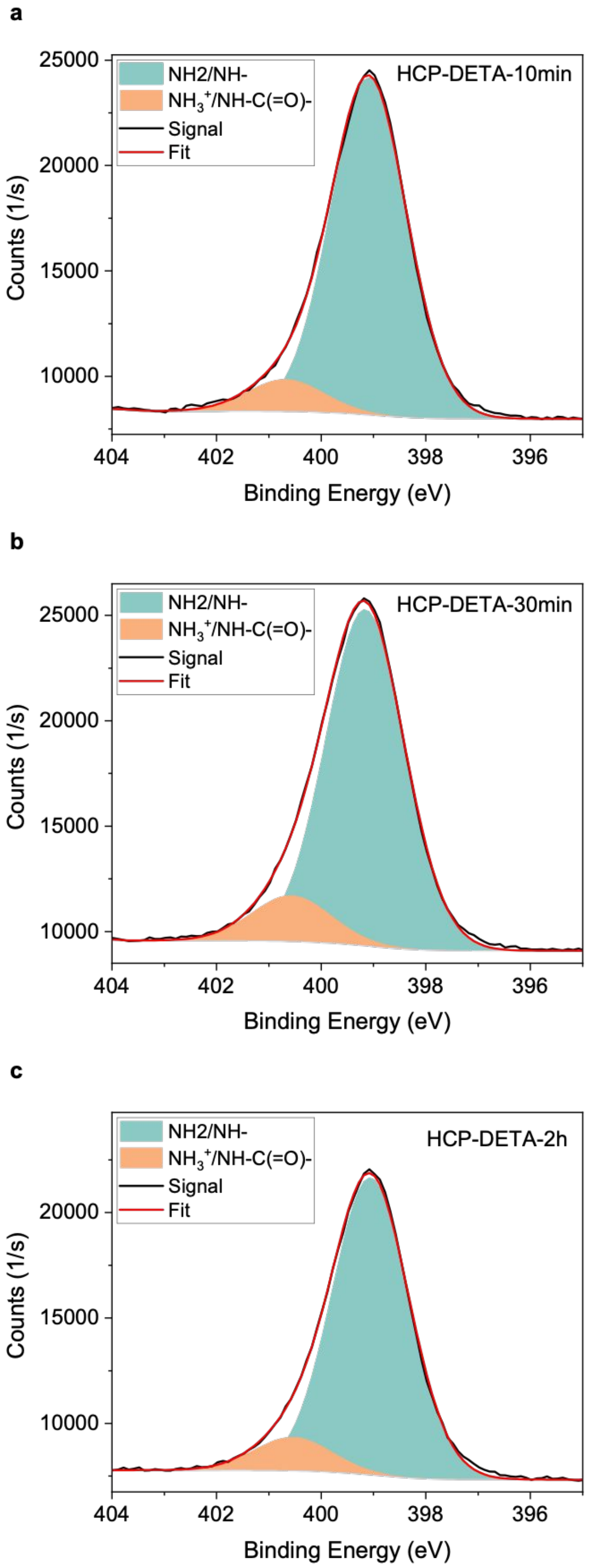

Figure S15. Deconvolution of the N *1s* XPS peak of a) HCP-DETA-10min, b) HCP-DETA-30min and c) HCP-DETA-2h. The two deconvoluted chemical environments are neutral amines with a peak around 399.2 eV and charged amines with a peak around 400.6 eV.

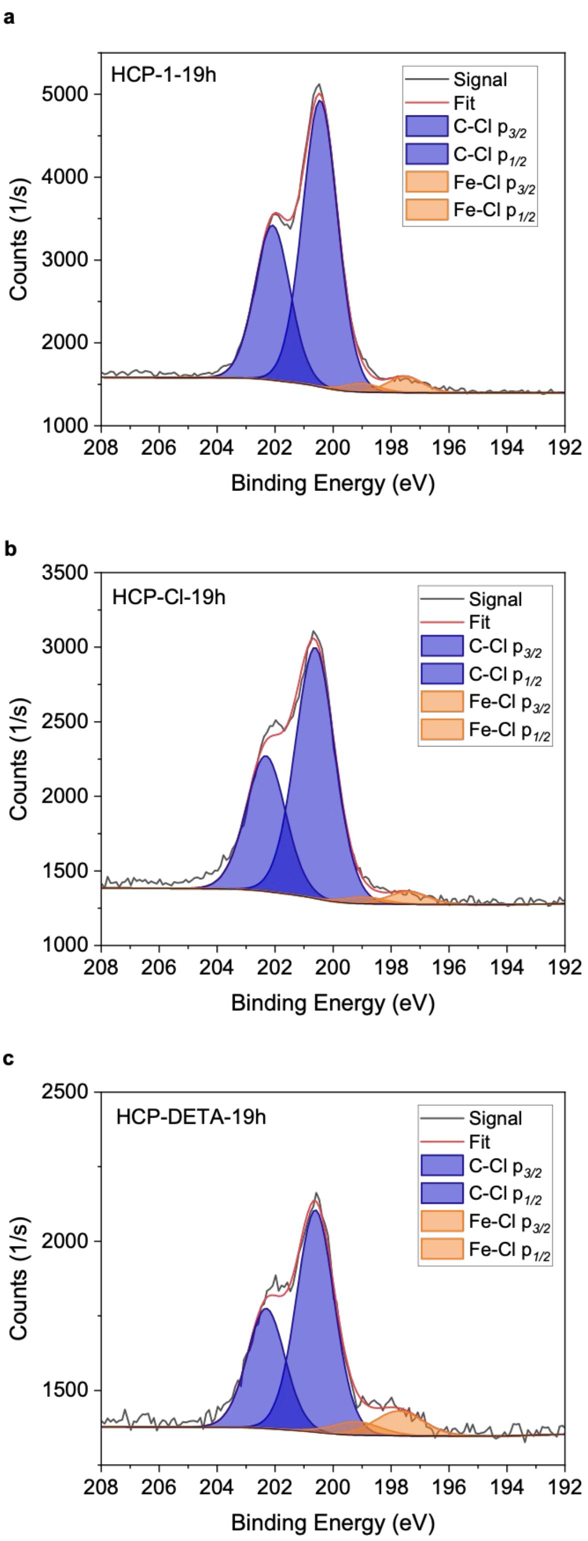

Figure S16: Deconvoluted chlorine XPS scans of a) HCP-1-19h, b) HCP-Cl-19h, and c) HCP-DETA-19h.

a

Covariance	σ	k_{LDF}
σ	4.092×10^{-9}	$-1.061 \times 10^{-13}\ s^{-1}$
k_{LDF}	$-1.061 \times 10^{-13}\ s^{-1}$	$1.937 \times 10^{-7}\ s^{-2}$

b

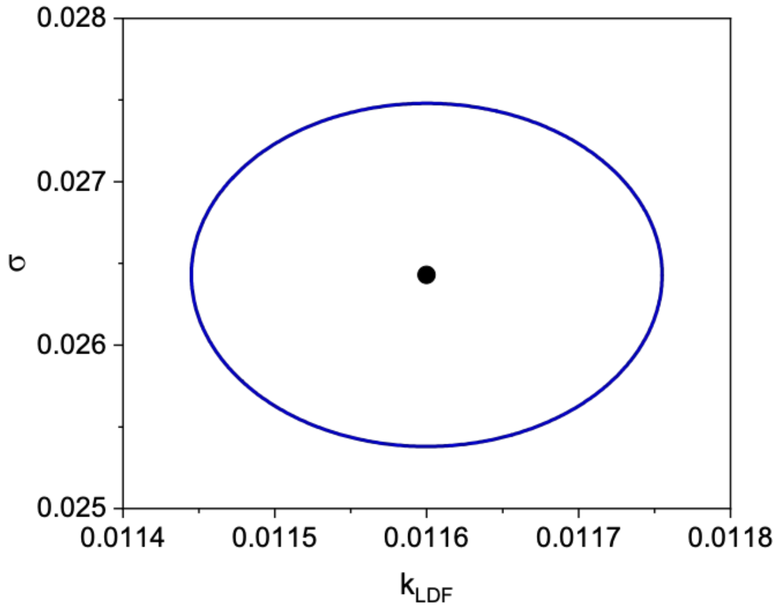

Figure S17: a Covariance matrix and **b** confidence ellipse of three repeats of the TGA uptake measurement at 400 ppm and a flowrate of 400 mL/min. The covariance matrix contains the variance terms of σ and k_{LDF} on the diagonal and the covariance between the two parameters on the off-diagonal.

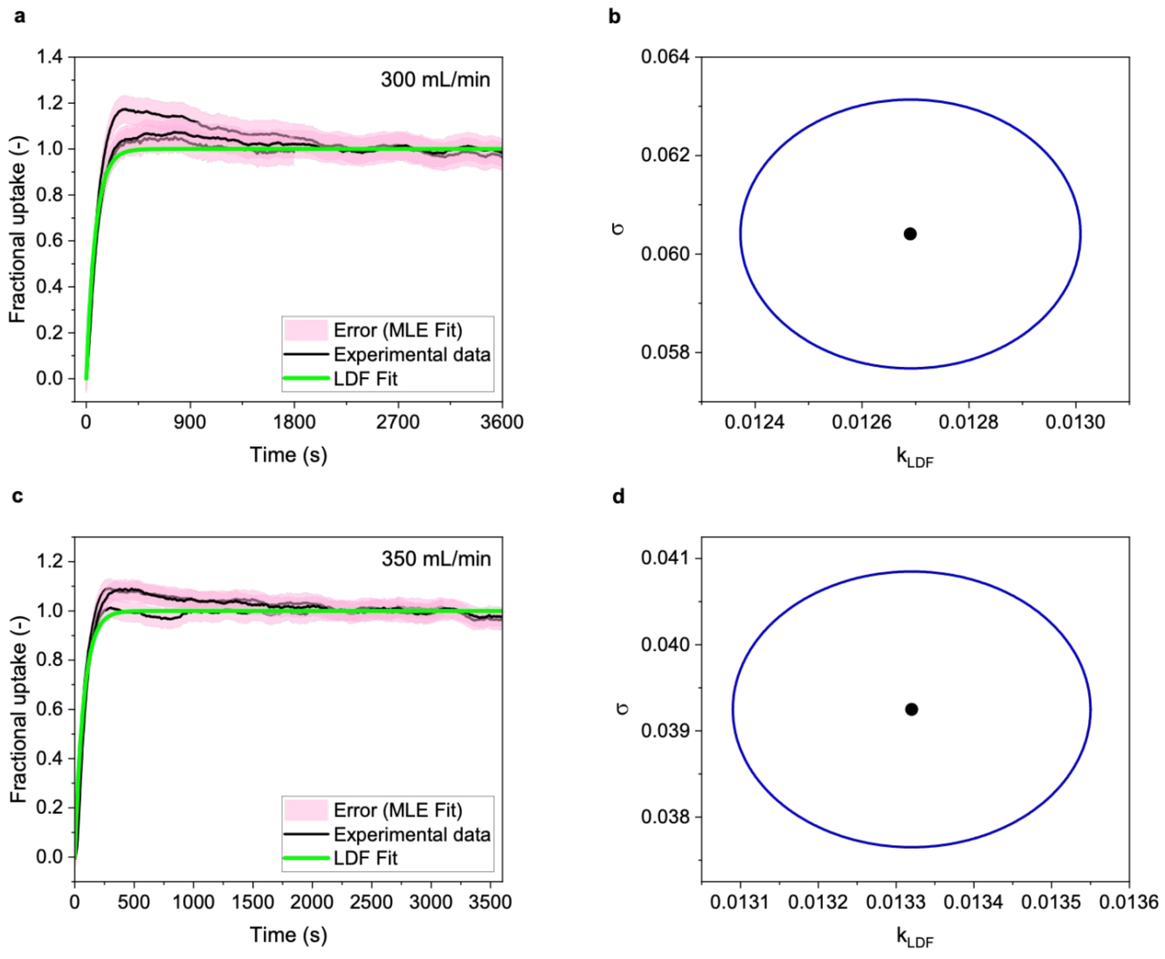

Figure S18: TGA uptake measurements as part of the flowrate study at two different flowrates with 400 ppm CO_2 in Helium atmosphere at 303 K. a) Uptake profiles of three repeats at a flowrate of 300 mL/min including the LDF fit. One of the measurements was interrupted after 1800 s while the other measurements were carried out for 3600 s. b) Confidence ellipse for the MLE fit at a flowrate of 300 mL/min. c) Uptake profiles of three repeats at a flowrate of 350 mL/min including the LDF fit. d) Confidence ellipse for the MLE fit at a flowrate of 350 mL/min.

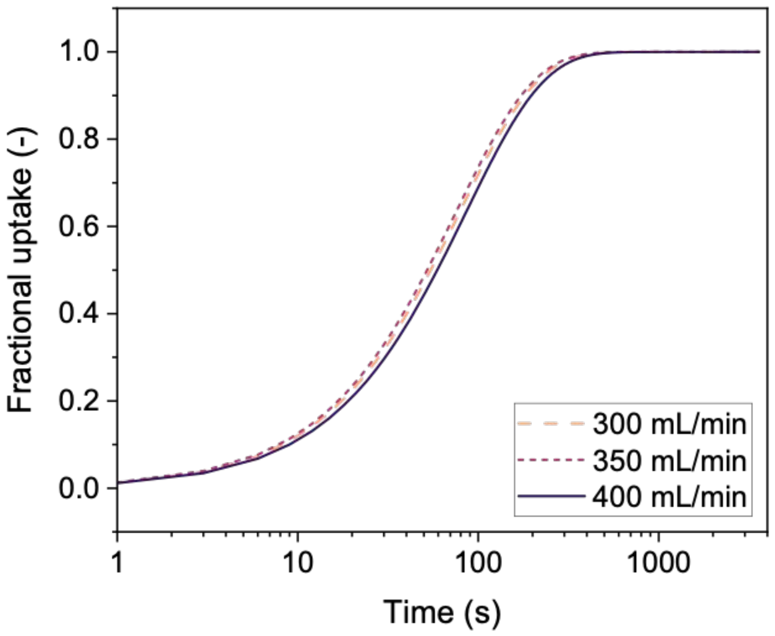

Figure S19: Comparison of the fitted LDF curves for flowrates of 300, 350, and 400 mL/min of HCP-DETA-10min in 400 ppm CO_2 in He at 303 K.

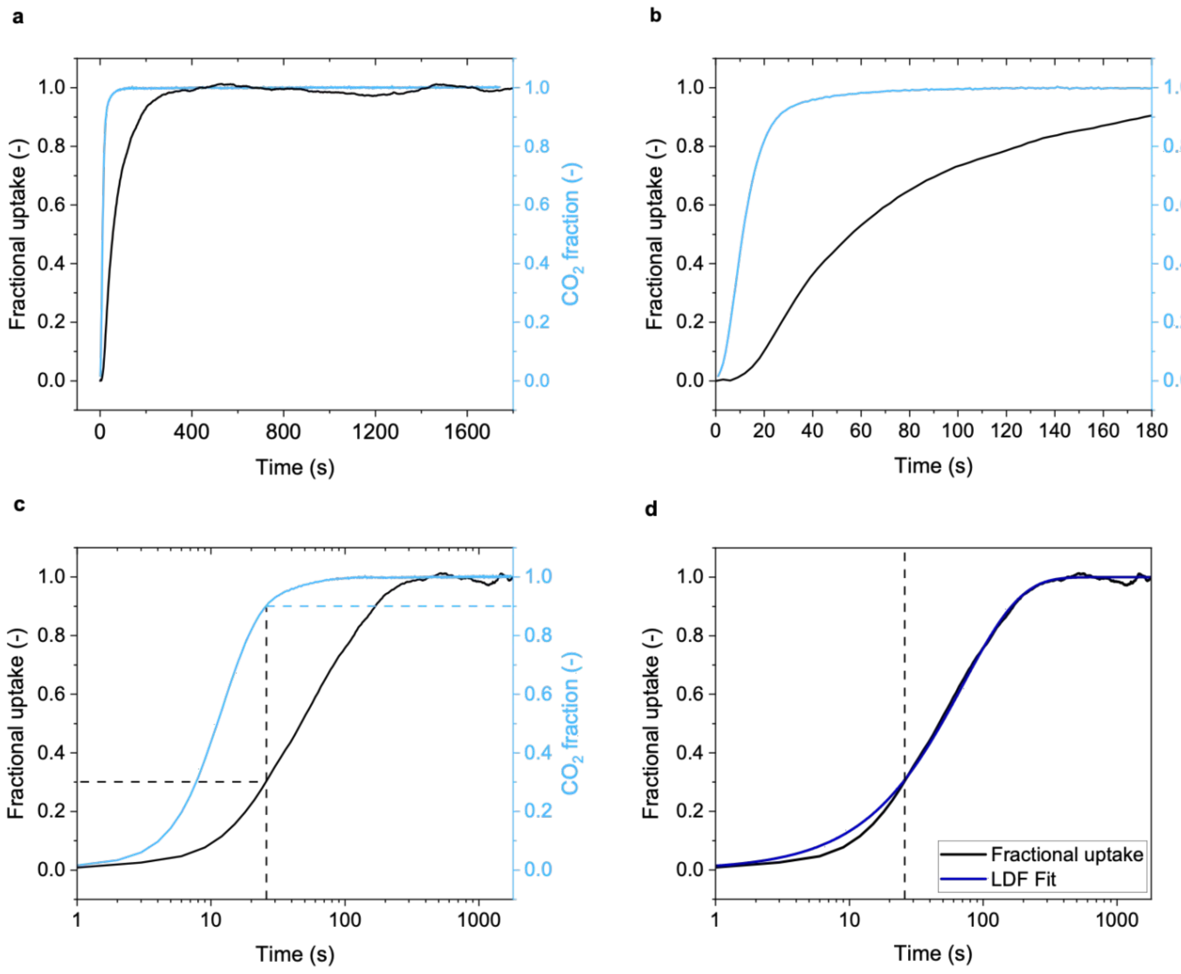

Figure S20: **a** Fractional of CO_2 at the outlet of the TGA furnace without sample (blue) and mass uptake (black) of CO_2 (400 ppm, 400 mL/min flowrate, 303 K) with the sample (HCP-DETA-10min) present plotted on an **a** linear (**b** zoomed) and **c** log10 axis. **d** LDF fit of a single TGA fractional uptake curve at 400 mL/min flowrate (400 ppm CO_2, 303 K). Plots **c** and **d** contain a horizontal line at t = 25 s corresponding to the time when an uptake of 30% and 90% of the final CO_2 concentration in the TGA analysis chamber were reached.

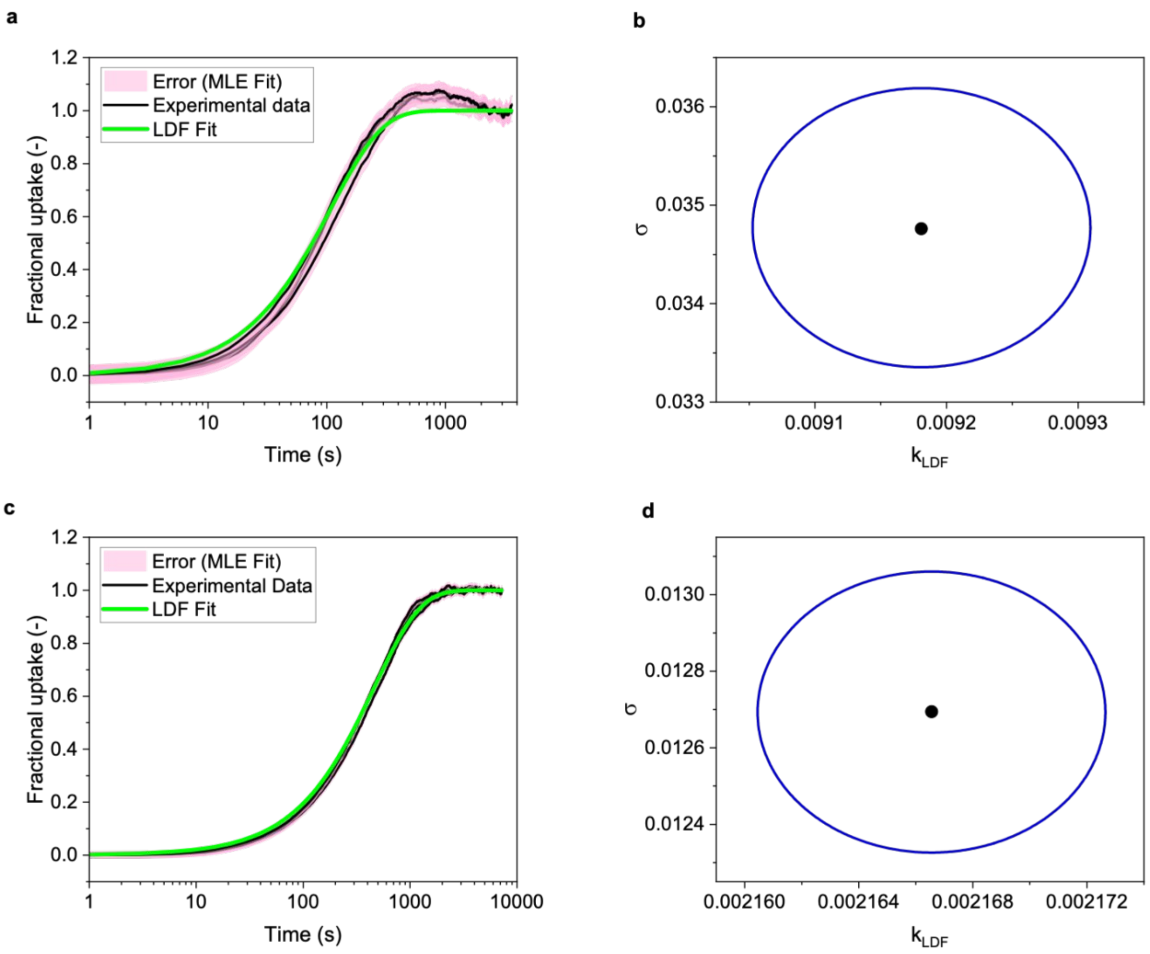

Figure S21: a) TGA uptake measurement of HCP-DETA-19h with 400 ppm CO_2 in Helium atmosphere at 303 K with 400 mL/min flow rate. b) Confidence ellipse for the MLE fit. c) TGA uptake measurement of Lewatit with 400 ppm CO_2 in Helium atmosphere at 303 K with 400 mL/min flow rate. d) Confidence ellipse for the MLE fit.

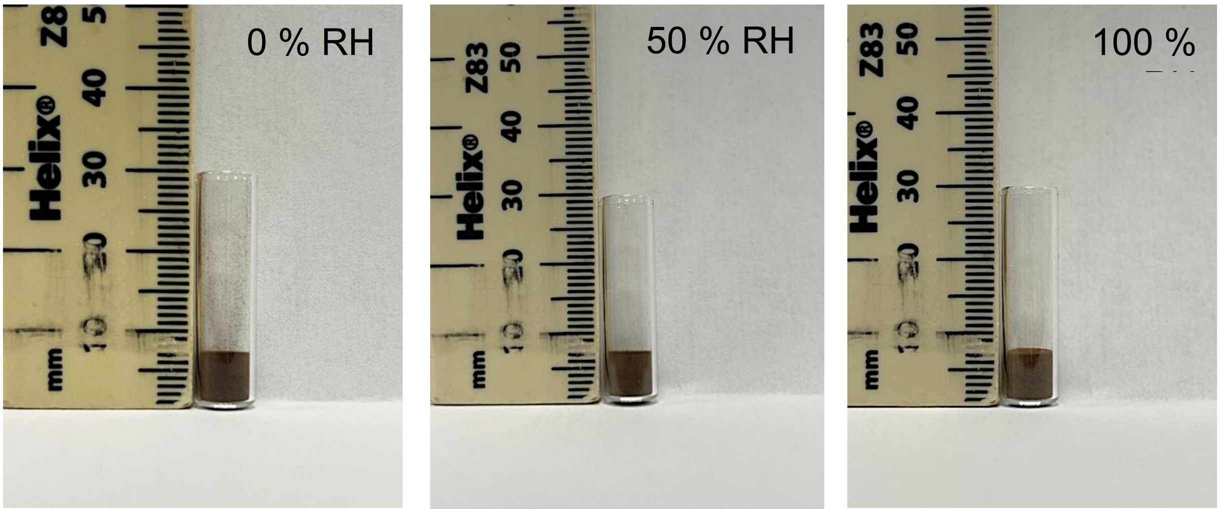

Figure S22: Swelling experiments of HCP-DETA-19h. The first image shows the pristine sample after drying in a vacuum at 60°C overnight (0% RH), the second image was taken after keeping the sample in a desiccator filled with a saturated NaNO3 solution overnight (50% RH), and the third image was taken after storing in a desiccator with DI water overnight (100% RH). All measurements were performed at room temperature.

Table S1: XPS Peak fitting parameters for the HCP-DETA peaks in Figs. 4b and S15. The area ratio is between the two peaks of the same material. L/G Mix is the percentage of the Lorentzian contribution to the Voigt profile. BE denoted the binding energy and carb./ammon. is short for the carbamate/ammonium peak.

Material	Peak	BE (eV)	Area (CPS*eV)	Area ratio	FWHM (eV)	L/G Mix
HCP-DETA-10min	1°/2° amine	399.09	29310	1.00	1.76	20.00%
HCP-DETA-10min	carb./ammon.	400.68	2780	0.09	1.76	20.00%
HCP-DETA-30min	1°/2° amine	399.16	30520	1.00	1.84	20.00%
HCP-DETA-30min	carb./ammon.	400.57	4150	0.14	1.84	20.00%
HCP-DETA-2h	1°/2° amine	398.76	26310	1.00	1.79	20.00%
HCP-DETA-2h	carb./ammon.	400.24	2970	0.11	1.79	20.00%
HCP-DETA-19h	1°/2° amine	399.24	12960	1.00	1.75	20.00%
HCP-DETA-19h	carb./ammon.	400.91	1810	0.14	1.75	20.00%

Table S2: Amine-contents and efficiencies of the four amine-functionalised HCPs. CO_2 uptake is given in mmol/g and the amine efficiency is calculated as the ratio of adsorbed CO_2 at 400 ppm over the theoretical uptake, assuming a 2:1 ratio of amine to CO_2 under dry conditions. The CO_2 uptake is determined at 298 K.

Material	N (at%)	N (mmol/g)	CO_2 uptake at 400 ppm (mmol/g)	Amine efficiency
HCP-DETA-10min	11.6	9.34	0.434	9.29 %
HCP-DETA-30min	11.1	8.94	0.320	7.16 %
HCP-DETA-2h	11.6	9.30	0.284	6.13 %
HCP-DETA-19h	7.4	5.9	0.125	4.3 %

Table S3: Comparison of HCP-DETA-10min to other amine-grafted adsorbents comprising silicas, metal oxides and COFs. Amine-impregnated adsorbents are not included in this table.

Material name	Material type	Amine-containing molecule	CO_2 uptake at 400 ppm (mmol/g)	T	Ref
SBA-15	Silica	APTES	0.3	298 K	[1]
SBA-15	Silica	AP-ESPP-B	0.42	298 K	[2]
TRI-$Mg_{0.55}$Al-a	Metal oxide	TRI	1.05	298 K	[3]
COF-609-Im	COF	TRPN	0.304	298 K	[4]
COF-999	COF	PEI (in-situ synthesis)	0.96	298 K	[5]
PPN-6-CH_2DETA	PPN	DETA	1.04	295 K	[6]
HCP-DETA-10min	HCP	DETA	0.43	298 K	This work
HCP-DETA-19h	HCP	DETA	0.13	298 K	This work

Abbreviations:

APTES: 3-aminopropyltriethoxysilane

AP-ESPP-B: N1-(3-Aminopropyl)-N1-(3-(triethoxysilyl)propyl)propane-1,3-diamine

TRI: 3-[2-(2-Aminoethylamino)ethylamino]propyl-trimethoxysilane

TRPN: tris(3-aminopropyl)amine

PEI: polyethyleneimine

PPN: Porous polymeric network

HCP: Hyper-crosslinked polymer

DETA: Diethylenetriamine

References

1. Wadi, B., et al. Evaluation of Moderately Grafted Primary, Diamine, and Triamine Sorbents for CO2 Adsorption from Ambient Air: Balancing Kinetics and Capacity under Humid Conditions. *Industrial & Engineering Chemistry Research*. **2021**, *60* (36), 13309-13317. 10.1021/acs.iecr.1c02416
2. Yoo, C.-J., S.J. Park, and C.W. Jones. CO2 Adsorption and Oxidative Degradation of Silica-Supported Branched and Linear Aminosilanes. *Industrial & Engineering Chemistry Research*. **2019**, *59* (15), 7061-7071. 10.1021/acs.iecr.9b04205
3. Zhu, X., et al. Modified layered double hydroxides for efficient and reversible carbon dioxide capture from air. *Cell Reports Physical Science*. **2021**, *2* (7). 10.1016/j.xcrp.2021.100484
4. Lyu, H., et al. Covalent Organic Frameworks for Carbon Dioxide Capture from Air. *J Am Chem Soc*. **2022**, *144* (28), 12989-12995. 10.1021/jacs.2c05382
5. Zhou, Z., et al. Carbon dioxide capture from open air using covalent organic frameworks. *Nature*. **2024**. 10.1038/s41586-024-08080-x
6. Lu, W., et al. Carbon Dioxide Capture from Air Using Amine-Grafted Porous Polymer Networks. *The Journal of Physical Chemistry C*. **2013**, *117* (8), 4057-4061. 10.1021/jp311512q
